# Fungus-growing insects host a distinctive microbiota apparently adapted to the fungiculture environment

**DOI:** 10.1038/s41598-020-68448-7

**Published:** 2020-07-24

**Authors:** Mariana O. Barcoto, Camila Carlos-Shanley, Huan Fan, Milene Ferro, Nilson S. Nagamoto, Mauricio Bacci, Cameron R. Currie, Andre Rodrigues

**Affiliations:** 1 Department of Biochemistry and Microbiology, São Paulo State University (UNESP), Rio Claro, SP Brazil; 2 Department of Biology, Texas State University, San Marcos, TX USA; 3 Department of Bacteriology, University of Wisconsin-Madison, Madison, WI USA; 4 Department of Plant Protection, São Paulo State University (UNESP), Botucatu, SP Brazil; 5 Center for the Study of Social Insects, São Paulo State University (UNESP), Rio Claro, SP Brazil

**Keywords:** Metagenomics, Metagenomics, Microbial ecology, Microbial ecology, Microbiome

## Abstract

Some lineages of ants, termites, and beetles independently evolved a symbiotic association with lignocellulolytic fungi cultivated for food, in a lifestyle known as fungiculture. Fungus-growing insects’ symbiosis also hosts a bacterial community thought to integrate their physiology. Similarities in taxonomic composition support the microbiota of fungus-growing insects as convergent, despite differences in fungus-rearing by these insects. Here, by comparing fungus-growing insects to several hosts ranging diverse dietary patterns, we investigate whether the microbiota taxonomic and functional profiles are characteristic of the fungiculture environment*.* Compared to other hosts, the microbiota associated with fungus-growing insects presents a distinctive taxonomic profile, dominated by Gammaproteobacteria at class level and by *Pseudomonas* at genera level. Even with a functional profile presenting similarities with the gut microbiota of herbivorous and omnivorous hosts, some differentially abundant features codified by the microbiota of fungus-growing insects suggest these communities occupying microhabitats that are characteristic of fungiculture. These features include metabolic pathways involved in lignocellulose breakdown, detoxification of plant secondary metabolites, metabolism of simple sugars, fungal cell wall deconstruction, biofilm formation, antimicrobials biosynthesis, and metabolism of diverse nutrients. Our results suggest that the microbiota could be functionally adapted to the fungiculture environment, codifying metabolic pathways potentially relevant to the fungus-growing insects’ ecosystems functioning.

## Introduction

Most of the organic carbon in land plants is stocked as lignocellulose^1^, a recalcitrant mesh constituted by biopolymers including cellulose, hemicellulose, pectin, and lignin^2,3^. For feeding on recalcitrant and indigestible lignocellulosic plant tissues, herbivorous animals rely largely on the association with symbiotic microorganisms, which mediates the use of otherwise non-accessible resources^4–7^. Besides metabolizing plant biomass components by hydrolysis and fermentation, the host-associated microbiota also assists the detoxification of plant-derived defensive secondary compounds^4,7,8^. A fascinating example of insect-microbial symbiosis for exploring recalcitrant plant biomass is observed in fungus-growing insects (FGI), which maintain lignocellulolytic fungi as crops^9^. The active maintenance of fungus crops, also known as fungiculture, evolved independently in three insect lineages^9^: ants in the subtribe Attina (Hymenoptera: Formicidae: Myrmicinae, “the attines”), which are strict to the New World^10,11^; beetles in the subfamilies Scolytinae and Platypodinae (Coleoptera: Curculionidae), which are predominantly found in tropical and subtropical ecosystems^12^; and termites in the subfamily Macrotermitinae (Isoptera: Termitidae), which occur in the Old-World tropics, mainly in Africa and Asia^13^.

The fungal lignocellulose-degrading capacity has been fundamental for the evolutionary success of the FGI symbiosis. For attine ants^14–16^, Macrotermitinae termites^17–19^, and ambrosia beetles^20^, the fungal symbionts metabolically convert recalcitrant plant biomass into highly nutritious and protein-enriched food, available to the farmer insect through mycophagy^21–24^. Bark beetles also obtain a nutritional supplementation for their phloem-based diet through fungal derived metabolites^25^. Fungiculture environments are also associated with a bacterial community that potentially regulates the symbiosis. For instance, some components of the bacterial microbiota from fungus-growing attine ants’ gardens are reported to fix atmospheric nitrogen^26^, being also metabolically capable of degrading lignocellulose and biosynthesizing amino acids and vitamins^27,28^. While bacteria found in ambrosia beetles galleries are considered secondary symbionts^29,30^, bacteria associated with bark beetles are able to produce antimicrobial compounds^31^ and to degrade the terpenes released by conifers as chemical defense^32,33^. In fungus-growing termites symbiosis, besides antimicrobial-producing bacteria potentially suppressing antagonistic fungi^34^, workers gut microbiota and the fungus comb microbiota aid in the comb continuous lignocellulose degradation^17,35^. Being associated with functionally herbivorous hosts suggests that these bacterial communities could take part in lignocellulose breakdown. However, it still remains unanswered by which means and to what extent the microbiota impacts nutrient cycling in different FGI symbioses.

**Figure 1:**
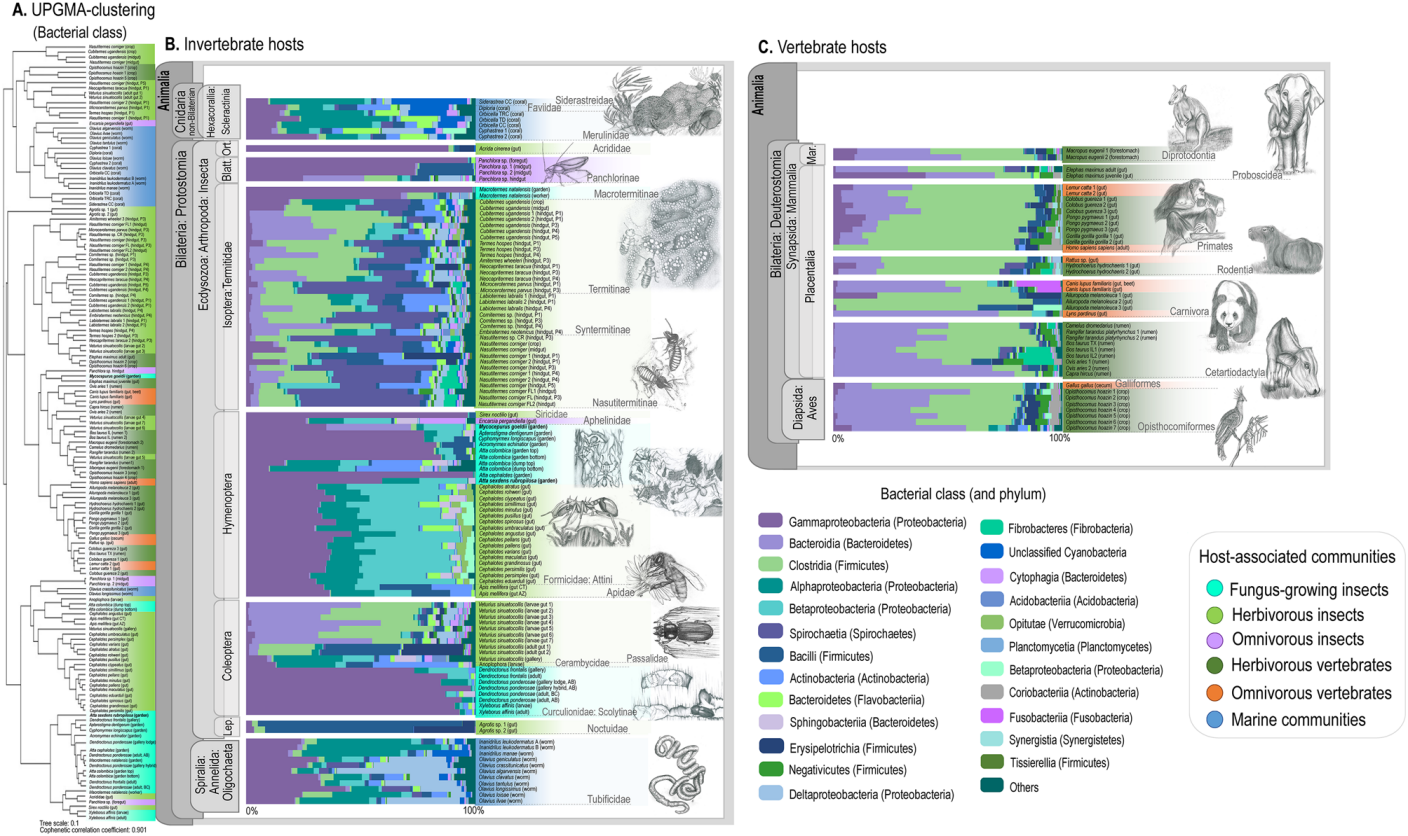
Microbiota composition at class level. Hosts are depicted according to their phylogenetic relationship and diet (detailed in Supplementary Table S1). Microbiota composition and similarity were estimated based on the normalized abundance of protein coding sequences taxonomically assigned at class level. (**a**) UPGMA-clustering estimated based on Bray–Curtis distances (Boot N = 10,000). (**b**) Relative abundance (%) of bacterial classes in the microbiota of invertebrate hosts. (**c**) Relative abundance (%) of bacterial classes in the microbiota of vertebrate hosts. Pencil drawings by Mariana O. Barcoto.

Environmental features particular to FGI ecosystems possibly impose selective pressures into the bacterial community, as despite differences in evolution, ecology and geographic distribution of their insect hosts, similarities in the microbiota composition point to taxonomic convergence^36^. Such taxonomic convergence could indicate functional convergence, since similar traits may independently evolve in multiple microbial lineages that are not necessarily phylogenetically related^37–39^. To define whether the microbiota of FGI exhibits a taxonomic and functional configuration characteristic of this environment, we compared these communities to several hosts ranging diverse diets (e.g., corals, marine worms, herbivorous and omnivorous insects and vertebrates; Fig. 1, Supplementary Table S1). For expanding the geographic distribution of FGI microbiota that are publicly available, we performed shotgun metagenome sequencing for microbial communities associated with fungus gardens of the attine ants *Mycocepurus goeldii* and *Atta sexdens rubropilosa*, both species widely distributed in Brazil^40,41^, which were grouped to a dataset from a previous study with FGI^36^. Comparing FGI to other hosts reveals a microbiota taxonomic composition that seems distinctive of attine ants’ fungus gardens, macrotermitine termites’ gut and fungus combs, galleries and gut of ambrosia and bark beetles. The FGI microbiota functional profile exhibit similarities with the gut microbiota of both herbivorous and omnivorous hosts, though some differentially abundant features codified by the FGI microbiota suggest these communities occupying microhabitats that could be characteristic of fungiculture. By suggesting the microbiota as functionally adapted to fungiculture environment, our findings reinforce the bacterial community as a structured and metabolically important feature of FGI ecosystems, possibly composing an essential part of FGI ecology.

## Results

### The microbiota of *Mycocepurus goeldii* fungus garden have a singular taxonomic composition within fungus-growing insects

We seek to expand the geographic distribution of the microbiota associated with FGI that are already available. Thus, we shotgun sequenced the metagenomes from fungus gardens of the lower attine *M. goeldii* and the higher attine *At. sexdens rubropilosa* from South America. Sequencing of the bacterial community obtained from *M. goeldii* fungus garden yielded 5.4 Gbp of raw sequence data (53,329,142 reads, Q30 = 91.22%). The bacterial community from *At. sexdens rubropilosa* fungus garden resulted in 6.7 Gbp of raw data (66,381,084 reads, Q30 = 85.96%). Reads of each library were assembled into metagenomes consisting of 249–364 Mbp of sequence data. Assembled contigs comprised good quality and length sequences (Supplementary Table S2). Fungus garden metagenomes from *M. goeldii* and *At. sexdens rubropilosa* are deposited at the IMG database, under the IMG Genome IDs 3300009856 and 3300009944, respectively.

**Figure 2:**
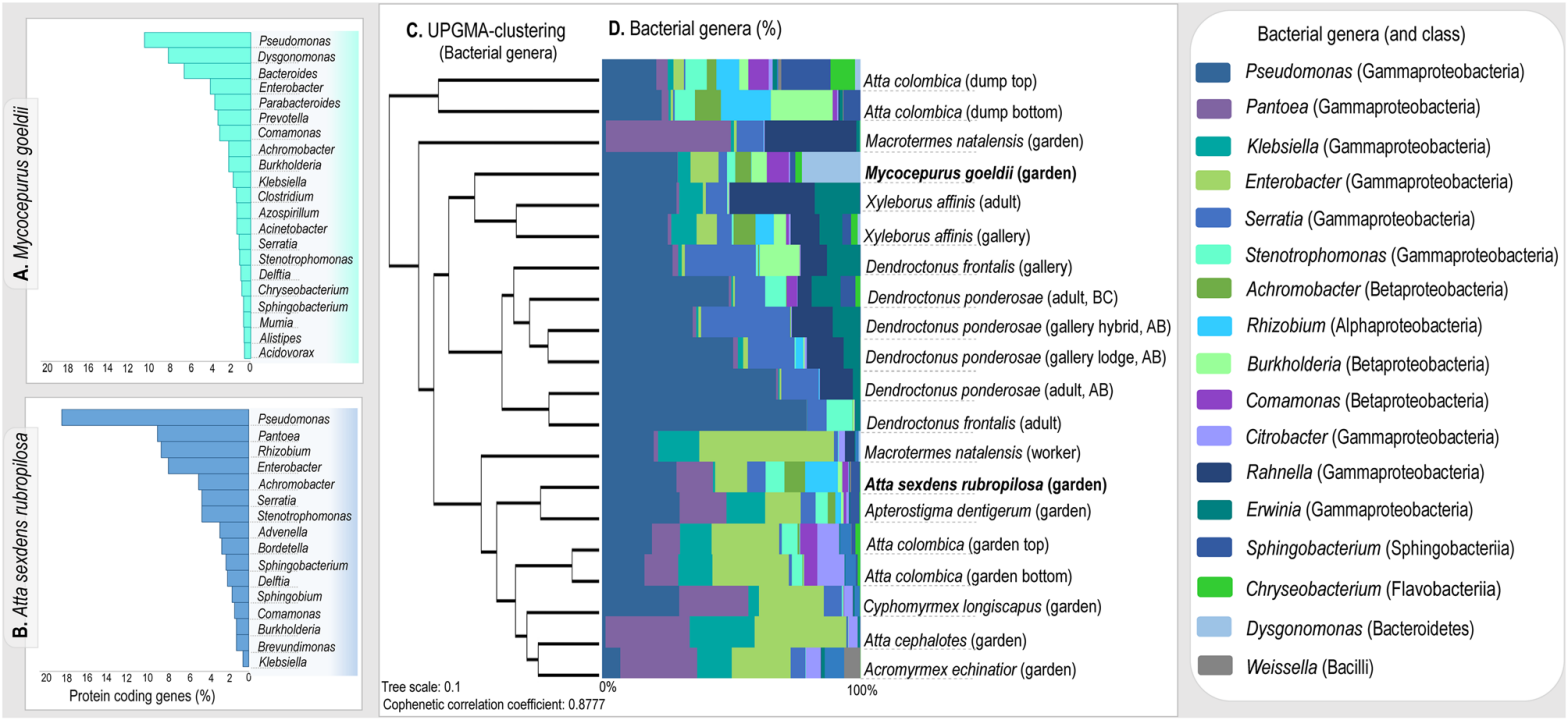
Most abundant bacterial genera of the microbiota associated with *Mycocepurus goeldii*, *Atta sexdens rubropilosa,* and other FGI. Sequences taxonomically assigned to the most abundant bacterial genera classified by COG functional categories for the microbiota of (**a**) *Mycocepurus goeldii* and (**b**) *Atta sexdens rubropilosa.* (**c**) UPGMA-clustering estimated based on Bray–Curtis distances (Boot N = 10,000) using the normalized abundance of protein coding sequences taxonomically assigned at genera level. (**d**) Relative abundance (%) of bacterial genera in the microbiota of FGI. The data presented in this figure also feature in the masters dissertation of M.O.B.^109^.

Gammaproteobacteria and Bacteroidia are similarly abundant for the microbiota of *M. goeldii* fungus garden, differing from the composition pattern dominated by Gammaproteobacteria that is observed in the FGI group (Fig. 1a). At genera level, *Pseudomonas*, *Dysgonomonas, Bacteroides, Enterobacter, Parabacteroides, Prevotella, Comamonas,* and *Burkholderia* are amongst the most abundant taxa in the bacterial community of *M. goeldii* fungus garden (Fig. 2a; Supplementary Figs. S1-S5). On the other hand, the microbiota of *At. sexdens rubropilosa* gardens follows the general taxonomic composition pattern found in other FGI, i.e., dominated by Gammaproteobacteria. Bacterial genera abundant in *At. sexdens rubropilosa* fungus garden include *Pseudomonas, Pantoea, Rhizobium, Enterobacter, Achromobacter, Stenotrophomonas* and *Serratia* (Fig. 2b; Supplementary Figs. S1-S5).

### The microbiota of fungus-growing insects have a particular taxonomic composition

Microbiota taxonomic composition was inferred by taxonomic assigning protein-coding sequences (PCS) through the “Phylogenetic Distribution of Genes” comparative tool of IMG^42^. At bacterial class level (identity percentage > 60%), FGI microbiota seems to share a particular microbiota composition, in most cases dominated by Gammaproteobacteria (Fig. 1a and b). When comparing the microbiota taxonomic composition between hosts with different diets and differing in phylogenetic distribution, UPGMA-clustering indicates the microbiota of FGI clustering separately from other hosts (Fig. 1a). Exceptions to this pattern may be observed in the microbiota composition of *M. goeldii* fungus garden (in which Gammaproteobacteria and Bacteroidia are similarly abundant) and the microbiota of *At. colombica* dump (that have Alphaproteobacteria and Actinobacteria as the most abundant taxa), both nesting into groups other than the FGI cluster (Fig. 1a). Even that the FGI cluster sits close to part of the herbivorous insects’ cluster, several bacterial classes significantly differed between them (White test, Bonferroni corrected *P* < 0.05; Supplementary Fig. S6A). For instance, while the relative abundance of Gammaproteobacteria is higher in the FGI microbiota, the relative abundance of Clostridia and Spirochaetia is higher in the gut microbiota of herbivorous insects (Supplementary Fig. S6A).

The FGI microbiota have the lowest diversity indices when compared to other hosts (Supplementary Fig. S7), exhibiting low taxa richness (Supplementary Fig. S7A) and diversity (Supplementary Fig. S7B), higher dominance (Supplementary Fig. S7C), and low evenness (Supplementary Fig. S7D). Marine communities (both the microbiota associated with corals and gutless worms) and herbivorous insects (particularly the Termitidae termites gut microbiota) present the highest taxa richness, diversity and evenness, as well as the lower dominance (Supplementary Fig. S7).

Even that taxonomic similarities within the FGI microbiota group are observed in higher hierarchical levels, their microbiota have particularities regarding genera-level composition. UPGMA-clustering based on sequences assigned to genera indicates three major groups of hosts’ microbiota (Fig. 2c), with the first cluster comprising *At. colombica* dump microbiota, which have *Pseudomonas, Stenotrophomonas*, *Rhizobium, Burkholderia,* and *Serratia* as the most abundant genera. *Pantoea, Serratia,* and *Rahnella* are the most abundant genera in *M. natalensis* garden’s microbiota, and this sample sits between the first and second clusters. The second cluster encompasses Scolytinae beetles’ microbiota, for which the majority of protein coding sequences is designated as *Pseudomonas, Serratia, Rahnella, and Erwinia. M. goeldii* microbiota sits close to this cluster, though presenting *Pseudomonas, Dysgonomonas, Pantoea,* and *Enterobacter* as the most abundant genera. The third cluster contains the microbiota of *M. natalensis* worker gut and Attini ants gardens, having sequences predominantly assigned as *Pseudomonas, Pantoea, Klebsiella,* and *Enterobacter* (Fig. 2d). Overall, the microbiota of FGI have a distinctive composition when compared to other hosts, being dominated by Gammaproteobacteria at class level and by *Pseudomonas* at genera level, showing low diversity and high dominance.

### Fungus-growing insects’ microbiota have a particular assemblage of CAZy-codifier bacterial groups

**Figure 3:**
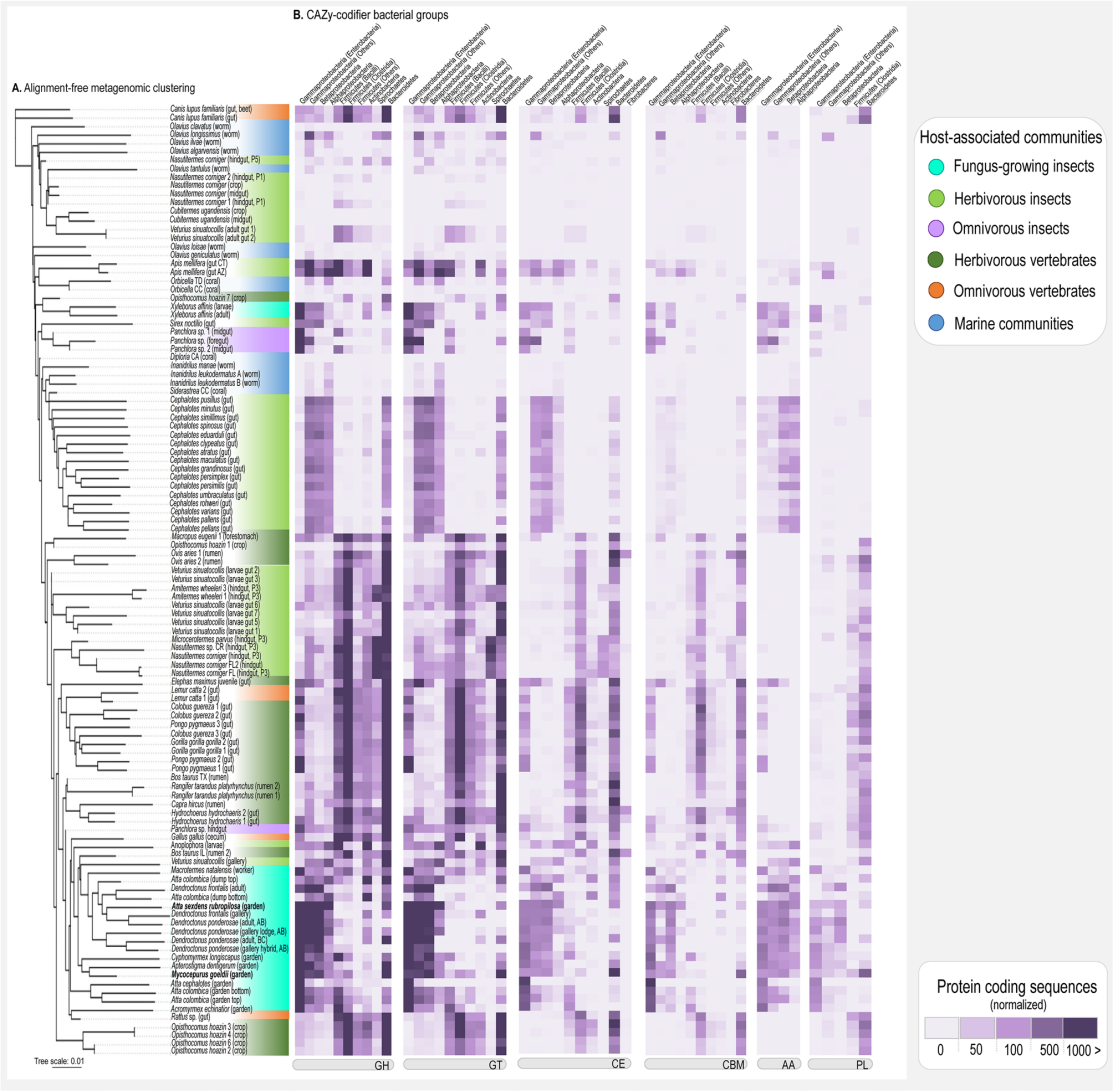
Metagenomic clustering and Carbohydrates-Active Enzymes (CAZy) taxonomic assignment**. **(**a**) Metagenomic clustering using alignment-free *k-*mer based approach (*k* = 15). (**b**) Class-level taxonomic classification of sequences assigned to each CAZy family, depicting the most abundant bacterial groups. Heatmaps constructed based on the normalized abundance of CAZy sequences taxonomically assigned.

The microbiota of FGI also group separately from the microbiota of other hosts by alignment-free *k-*mer based approach for metagenome clustering (Fig. 3a). This clustering reflects the particular CAZy-codifier microbiota of FGI (Fig. 3b), assigned mainly as Gammaproteobacteria (Enterobacteria and Others), Betaproteobacteria, and Alphaproteobacteria (Supplementary Figs. S8 and S9). A similar pattern occurs in the cluster comprising the microbiota associated with the omnivorous *Panchlora* sp. cockroaches, the herbivorous *Sirex noctilio* wasp, and the fungus-growing beetle *Xyleborus affinis*. Though presenting a similar CAZy-codifier microbiota dominated by Gammaproteobacteria (Enterobacteria and Others), and Firmicutes (Bacilli), this cluster is separated from other FGI.

Herbivorous insects clustered in four main groups (Fig. 3, Supplementary Fig. S9): I) Cluster encompassing the microbiota of the initial segment of *Nasutitermes corniger* and *Cubitermes ugandensis* termites gut, as well as the microbiota of adult *Veturius sinuatocollis* beetles, that does not present a particular CAZy-codifying microbiota. II) Cluster containing *Apis mellifera* gut microbiota, that have Gammaproteobacteria (Others and Enterobacteria), Firmicutes (Bacilli), Actinobacteria, and Alphaproteobacteria as the most abundant CAZy-codifier members; III) *Cephalotes* ants cluster, in which CAZy-codifier members are Gammaproteobacteria (Others), Betaproteobacteria, Alphaproteobacteria, and Bacteroidetes. IV) Clustering of the gut microbiota of the termites *Amitermes wheeleri, Microcerotermes parvus, N. corniger* hindgut and the gut of *V. sinuatocollis* larvae, that have as the most abundant CAZy-codifier members Firmicutes (Bacilli and Clostridia), Spirochaetes, and Bacteroidetes.

Herbivorous vertebrates also clustered separately, forming three main groups (Fig. 3, Supplementary Fig. S9): I) *Macropus eugenii* and *Ovis aries* gut cluster, for which Firmicutes (Bacilli and Clostridia) and Bacteroidetes are the most abundant groups. II) For the cluster that comprises the gut microbiota of Primates and Rodentia hosts, the most abundant CAZy-codifier members are Firmicutes (Bacilli and Clostridia), Bacteroidetes, and Gammaproteobacteria (Enterobacteria). III) The cluster containing *Opisthocomus hoatzin* crop microbiota presents higher abundances of Firmicutes (Bacilli and Clostridia) and Bacteroidetes.

Two general patterns were observed for the taxonomically assigned CAZy sequences of gut microbiota of omnivorous vertebrates (Fig. 3, Supplementary Fig. S9). First, the gut microbiota of *Canis lupus familiaris* and *Rattus* sp. in which Firmicutes (Bacilli and Clostridia) and Bacteroidetes are the most abundant CAZy-codifier members. Second, the gut microbiota of *Lemur catta* that presents higher abundance of Firmicutes (Bacilli and Clostridia), Bacteroidetes, and Gammaproteobacteria (Enterobacteria), and clustered with the Primates group. The marine bacterial communities have low relative abundance of CAZy-annotated sequences, not presenting a particular CAZy-codifying microbiota. In general, when comparing hosts with different diet and lifestyle, the CAZy-codifier community dominated by Gammaproteobacteria seems to be a characteristic feature of the microbiota associated with FGI.

### The microbiota of fungus-growing insects codify diverse metabolic pathways apparently adapted to fungiculture environment

**Figure 4:**
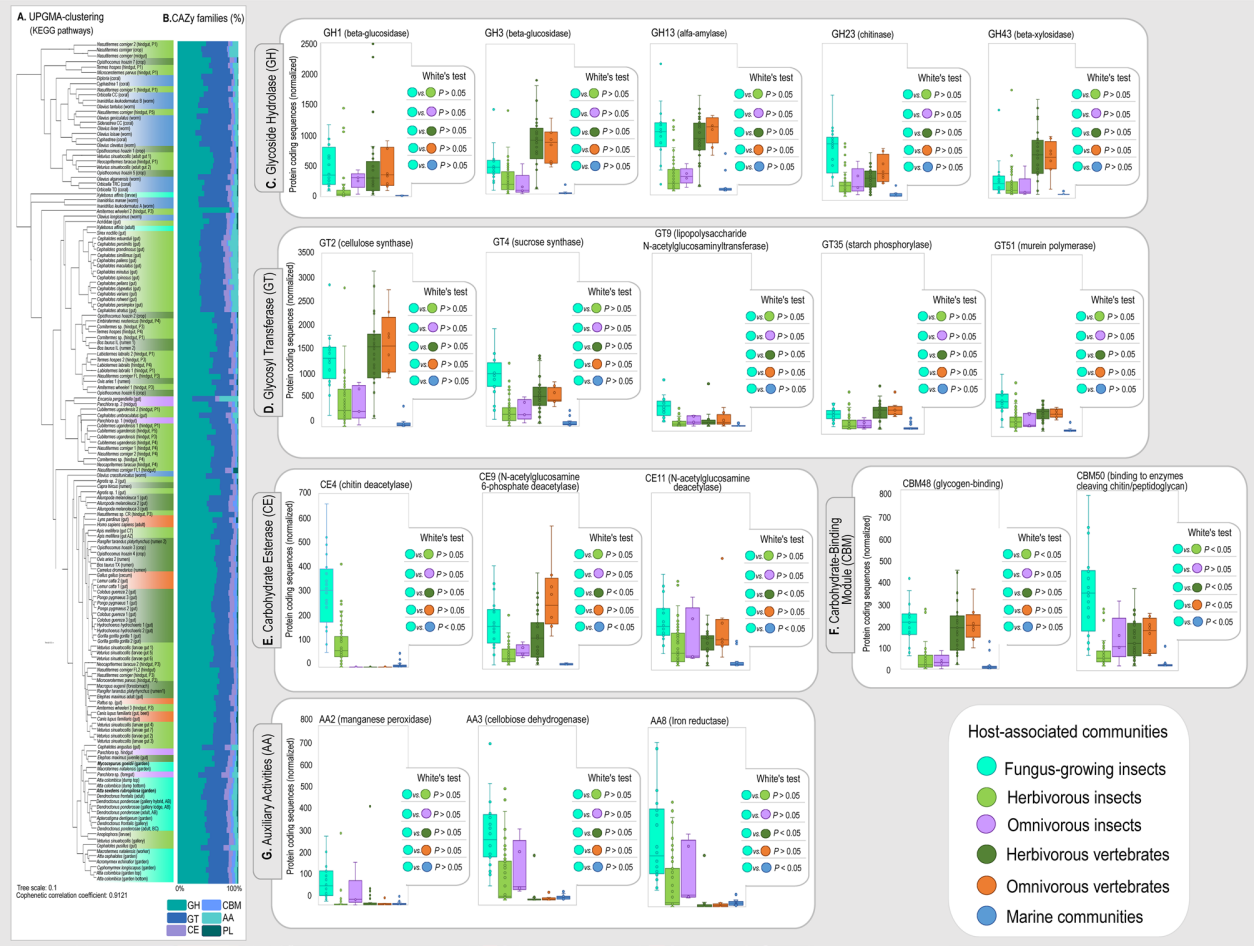
Functional profile estimated via KEGG pathways and CAZy families (**a**) UPGMA-clustering estimated based on Bray–Curtis distances (Boot N = 10,000) using the relative abundance of KEGG assigned sequences. (**b**) Relative abundance of protein-coding sequences assigned as CAZy. Box plots calculated based on the normalized abundance of the CAZy families abundantly codified by the microbiota of FGI. Comparisons between host groups were determined using the White’s test^105^ (**c**) Most abundant GH (glycoside hydrolases). (**d**) Most abundant GT (glycoside transferases). (**e**) Most abundant CE (carbohydrate esterase). (**f**) Most abundant CBM (carbohydrate-binding modules). (g) Most abundant AA (auxiliary activities). Because of the low number of protein coding sequences annotated as PL (polysaccharide lyases, PCS < 100) in the FGI microbiota, these functions are not depicted.

Functional profile of the hosts’ microbiota was predicted through KEGG pathways (via KO terms) and CAZy families. UPGMA-clustering based on the normalized number of PCS assigned to KEGG pathways at a general level have not evidenced well delimited clustering patterns according to diet or host phylogeny (Fig. 4a). Most samples of the microbiota associated with FGI tended to sit close to one another in UPGMA-clustering, and the KEGG profile of the FGI cluster shares features with the microbiota associated with herbivorous and omnivorous hosts (both insects and vertebrates; Fig. 4, Supplementary Figs. S10-S18). However, compared to other hosts the microbiota associated with FGI have a higher abundance (White test, Bonferroni corrected *P* < 0.05) of functions assigned to: carbohydrate pathways related to glyoxylate and dicarboxylate, butanoate, and propanoate metabolism (Supplementary Fig. S10); amino acid pathways related to tyrosine, glutathione, arginine and proline, phenylalanine, tryptophan, valine, leucine and isoleucine metabolism (Supplementary Fig. S11); energy pathways related to sulfur and nitrogen metabolism (Supplementary Fig. S12); glycan pathways related to lipopolysaccharide biosynthesis (Supplementary Fig. S13); lipid pathways related to fatty acid degradation and biosynthesis of unsaturated fatty acids (Fig. S14); cofactors and vitamins pathways related to biotin metabolism and terpenoid-quinone biosynthesis (Supplementary Fig. S15); terpenoids and polyketides pathways related to geraniol, limonene and pinene degradation, and biosynthesis of siderophore nonribosomal peptides (Supplementary Fig. S16); secondary metabolism pathways related to tropane, piperidine, pyridine alkaloid, and isoquinoline alkaloid biosynthesis (Supplementary Fig. S17); and xenobiotics pathways related to benzoate degradation (Supplementary Fig. S18).

**Figure 5:**
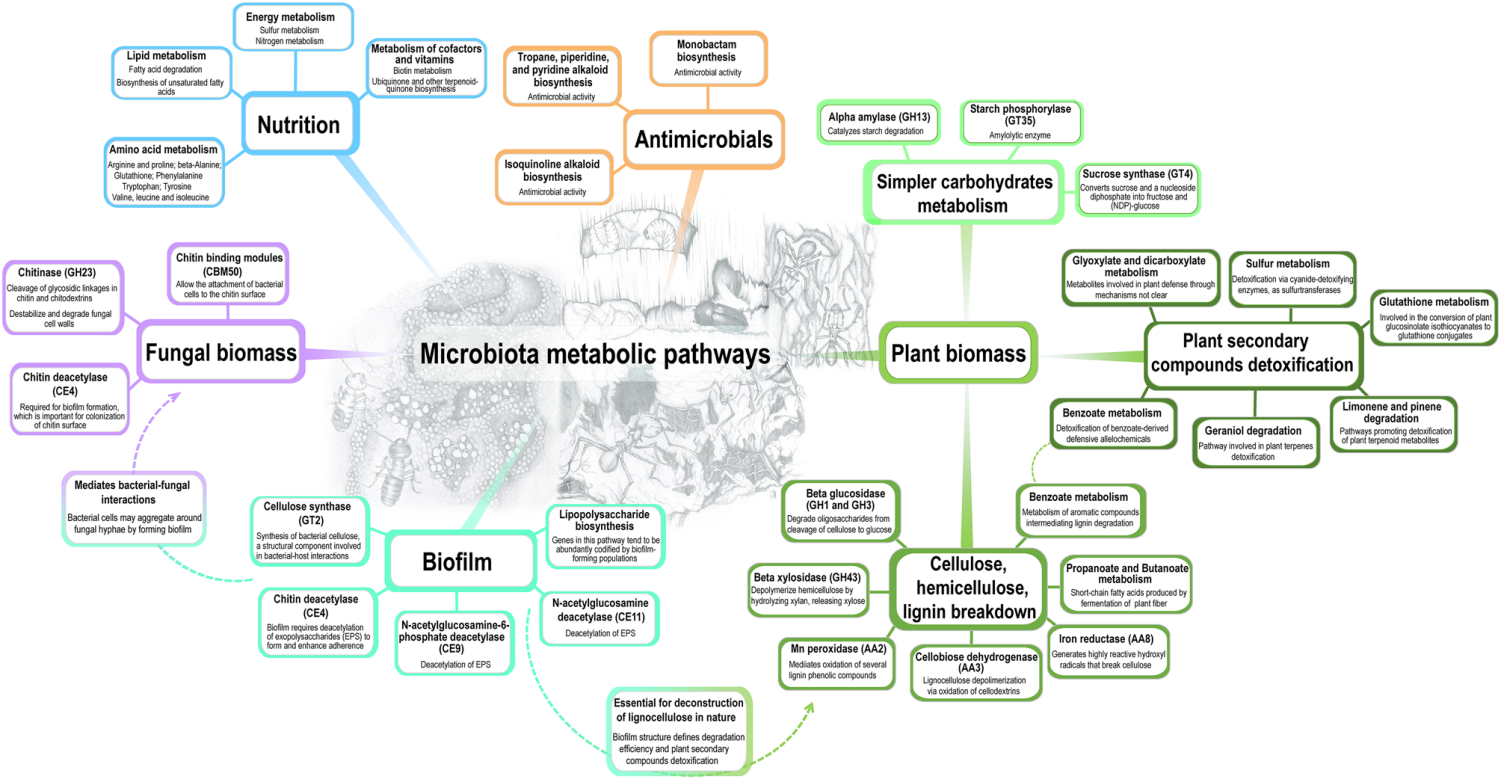
Differentially abundant features codified by the fungus-growing insects’ microbiota. Summary of abundant CAZy families and KEGG pathways that are more abundantly codified by the FGI microbiota when compared to other hosts (White test, Bonferroni corrected *P* < 0.05)^105^. Possible metabolic roles for these functions were speculated according to the literature (Supplementary Table S4). Diverse studies suggest these functions participating in plant biomass metabolism, biofilm formation, fungal biomass metabolism, general nutrition, and antimicrobials biosynthesis. Abundance and statistic comparisons of each CAZy family and KEGG pathway may be found at Fig. 4, and Supplementary Figs. S10-S18. Ant gardens, termite combs, and beetle galleries depicted at the center exhibit characteristic structures deriving from the metabolism of plant biomass. Pencil drawings by Mariana Barcoto.

At a general level, the relative abundance of PCS annotated for CAZy families reveals that the microbiota of FGI tend to exhibit a higher relative abundance of PCS classified as GH and GT, followed by AA and CE (Fig. 4b), a pattern that is also observed for the gut microbiota of the herbivorous *Cephalotes* ants, *Anoplophora* larvae gut, *V. sinuatocollis* beetle’s gallery, and for the omnivorous *Panchlora* sp. cockroach. Other herbivorous insects, herbivorous vertebrates, and omnivorous vertebrates have a higher proportion of PCS assigned to GH family, followed by GT and CBM. Within CAZy families, the most abundant CAZy functions codified by the microbiota associated with FGI include: GH 1, GH 3, GH 13, GH 23, GH 43, GT 2, GT 4, GT 9, GT 35, GT 51, CE 4, CE 9, CE 11, CBM 48, CBM 50, AA 2, AA 3, AA 8 (Fig. 4c-g; Supplementary Table S3). In summary, though having some functional overlapping with the gut microbiota of herbivorous and omnivorous hosts, the FGI microbiota differentially codify functions in pathways related to lignocellulose breakdown, detoxification of plant secondary metabolites, metabolism of simple sugars, fungal cell wall deconstruction, biofilm formation, antimicrobials biosynthesis, and diverse nutrient cycling routes (Fig. 5).

## Discussion

Besides obtaining nutrients through a symbiotic association with fungi, FGI are associated with a bacterial community physiologically important for the insect-host lifestyle^17,27,28,43^. Even though FGI differ regarding geographic distribution, evolutionary history, and fungal taxa maintained as crops^9^, marked similarities in microbiota taxonomic composition at higher hierarchical levels (e.g. phylum and class) support a convergence of the host-microbiota association^36^. At class level, FGI colonies and galleries seem to assemble a microbiota particular to these environments, having the Gammaproteobacteria as the most abundant group (Fig. 1, Supplementary Fig. S1) and low class-diversity (Supplementary Fig. S7). At genera level, despite particularities regarding the relative abundance of specific genera, *Pseudomonas, Pantoea, Klebsiella, Enterobacter,* and *Serratia* are relatively abundant for fungus-growing ants, termites, and beetles (Fig. 2c,d), and could be considered part of the FGI core microbiota^36^. The microbiota of *M. goeldii* fungus-garden tend to differ from other FGI, by exhibiting different patterns of taxonomic composition including Gammaproteobacteria and Bacteroidia as equally abundant classes (Fig. 1a), and *Dysgonomonas* among the most abundant genus (Fig. 2a and c). However, based on the small amount of metagenomic data available for lower attine ants, we are not able to determine whether the taxonomic composition of *M. goeldii* could also extend to other lower attines. Even so, considering the diversity of lower attine ants^11^, it is also possible that different ant species could host taxonomically diverse microbiota that would include different dominant taxa. Therefore, having a particular taxonomic composition could not be an exclusivity of the *M. goeldii* microbiota, or yet, could be a feature commonly spread throughout the lower attines group.

Gammaproteobacteria-enriched communities of FGI codify for diverse carbohydrate-active enzymes (Fig. 3) potentially related to plant biomass deconstruction, biofilm formation, and fungal biomass metabolism (Fig. 4). Some of these features overlap with herbivorous and omnivorous hosts, indicating functional similarities with these environments at a certain extent (Fig. 4, Supplementary Figs. S10-S18). Together, abundantly codified CAZy families and KEGG pathways may reflect functions important for the FGI microbiota metabolism, suggesting the community participating in lignocellulose breakdown, detoxification of plant secondary metabolites, metabolism of simple sugars, fungal cell wall deconstruction, biofilm formation, antimicrobials biosynthesis, and diverse nutrient cycling routes (Fig. 5). Fungus gardens, combs, and galleries are considered to act as aerobic external guts, metabolizing recalcitrant plant biomass into simpler carbohydrates^21–24^, that become available to the insect host^17,20,44–46^. As the most abundant bacterial genera in these communities are aerobes/facultative anaerobes (Fig. 2)^47,48^, environmental aerobic conditions apparently impact the microbiota composition. Because oxygen is required for lignin breakdown^49^, aerobic conditions could favor lignin depolimerization by microorganisms codifying ligninolytic enzymes. The FGI microbiota has been suggested as part of plant biomass metabolism^17,20,27,35,43^, though the mechanisms and pathways for this integration remain to be further explored. By potentially metabolizing complex plant components and degrading toxic compounds (Fig. 5), members of the FGI microbiota could mirror roles of an herbivorous gut microbiota fundamental for herbivorous hosts nutrition^50–54^.

Fungiculture environments could also favor groups of microorganisms degrading plant fibers via pathways alternative to those commonly codified by herbivorous gut microbiota (for instance, those able to metabolize lignocellulose in aerobic conditions that are not found in the gut; Figs. 3 and 4)^50^. Independent of the taxonomic composition, these microbial communities would have similar functional groups (Fig. 3)^55–58^ exploiting plant-derived resources. Plant cell wall deconstruction in FGI symbiosis could sustain complimentary roles of the fungal symbiont and the associated microbiota, resulting in a multipartite metabolism of lignocellulose. CAZy families codified and/or expressed by the fungal symbiont of FGI tend to target complex polysaccharides (as cellulose, hemicellulose, pectin, and starch) by a different enzymatic repertoire^15,17,59^ than that codified by the bacterial community (Fig. 5). By assembling the plant biomass degradation in tandem, the fungal-microbiota association could efficiently metabolize lignocellulose even whether none of the organisms codify the complete enzymatic pathway^50,60,61^. Also crucial for maintaining a healthy fungiculture is detoxifying plant secondary compounds, as several of these metabolites (specially terpenoids) are harmful for both the insect and fungal symbiont^62,63^. The FGI microbiota may have an important role in detoxifying plant metabolites^43^, which could select microbial members able to metabolize these toxic compounds, influencing the microbiota composition. Members of the FGI core microbiota including *Pseudomonas, Rahnella, Serratia, Burkholderia*^43,64^, and *Stenotrophomonas*^65^, are reported to detoxify plant compounds, which could also be accomplished in the fungiculture environment. As for the gut microbiota of herbivorous hosts, the FGI microbiota could degrade plant secondary compounds into innocuous metabolites that would enter into the host’s nutritional pathways^53^.

Differentially abundant functions related to biofilm formation (Fig. 5) could indicate these pathways influencing the FGI microbiota lignocellulolytic activity^66–68^ as reported for other plant biomass-degrading communities^53,69^. Communities embedded in biofilm matrix optimize lignocellulose breakdown by retaining and accumulating degradative enzymes and depolymerization products, allowing the attachment to the plant substrate and permanence at the hydrolysis site, supporting syntrophic associations between microorganisms and thus forming trophic chains required for degradation of plant polymers^51,66–68^. Host-associated biofilm-forming communities not only detoxify plant secondary compounds through sorption of toxins into the matrix^53^, but also retain nutrients and metabolic products that become available for assimilation by the community and the host^51,66,67^. Nutritional support to the fungal symbiont has already been suggested as a role of the FGI microbiota^26–28^, which could involve pathways related to nitrogen, sulfur, amino acids, lipids, and vitamins metabolism (Fig. 5; Supplementary Figs. S11, S12, S14, S15). Investigating nutrient-based interactions could reveal fungal-microbiota integrated networks for nutrient cycling important for FGI ecosystem functioning. For instance, bacteria in some plant decomposer communities make nitrogen available to fungi while receiving labile carbon compounds in exchange^70,71^, and similar networks could be operating in the fungiculture environment^26^. Moreover, functions codified by the FGI microbiota suggest the attachment to fungal cell walls, possibly via biofilm formation (Fig. 5). In the fungiculture scenario, biofilms could mediate fungal-microbiota interactions^70, 72^ including bacterial mycolytic activity, as pathways related to the metabolism of chitin may reflect the populations obtaining nutrients from hyphae^63–65,73–77^. This opens the possibility of populations within the FGI microbiota participating in fungal biomass turnover by consuming fungal nutrients from old and metabolically inactive portions of fungus gardens, combs, and galleries. Alternatively, bacterial populations could act as commensals throughout the system, obtaining resources from hyphae (as carbohydrates, protein, lipids)^73^ and exudates (low molecular weight metabolites), but not leading to harmful interactions^77^. It is curious to observe that communities living in ectomycorrhiza mycosphere (i.e., the region within and surrounding hyphae)^78^ tend to be dominated by *Pseudomonas* species able to metabolize fungal exudates^79,80^, raising questions on the possibility of such interactions to occur in fungiculture.

It also remains to be investigated the likelihood, extent, and metabolic outcomes of interactions occurring among bacterial populations within the FGI microbiota^81^. For instance, the abundance of pathways related to antimicrobials biosynthesis (Fig. 5) points to several viable interactions, including either competition among bacterial populations, between the microbiota and the fungal symbiont, or cooperation for defending the symbiosis against pathogens^81,82^. Also insightful would be to analyze the distribution, diversity, and stability of bacterial populations across the gradient of nutrients that derive from plant biomass metabolism by the fungal symbiont^82–84^. Overall, features abundantly codified by the FGI microbiota may reflect a multiplicity of microhabitats distinctive of fungiculture, deriving from an assemblage of conditions including the availability of raw plant biomass, simpler carbohydrates and lignin-derivatives resulted from fungal metabolism, fungal biomass, and aerobic environments. Merging these conditions could result in niches (i.e., ecological role and space occupied by a microorganism within a community) particular to fungiculture, favoring microorganisms able to explore these resources and ultimately defining the microbiota composition^85–87^. Such environmental particularities shaping the microbiota could result in the low class diversity and high dominance observed for this group (Supplementary Fig. S7). Our findings highlight the complexity and heterogeneity of the FGI microbiota metabolic pathways, suggesting the microbiota as possibly adapted to the fungiculture environment. Such perspective emphasizes the need to further investigate FGI ecosystems, not only for their potential to codify for natural products^88,89^ and biotechnologically important enzymes^90^, but also to unveil the ecological relevance of microbiota-fungal metabolic networks fundamental to FGI evolutionary success.

## Methods

### Fungus-garden sampling

We expanded the dataset of FGI microbiota by sequencing the microbial community from fungus gardens of the lower attine *M. goeldii* and the higher attine *At. sexdens rubropilosa*. Fungus gardens, ants, and brood from visibly healthy colonies of *At. sexdens rubropilosa* and *M. goeldii* were collected from nests near Botucatu, São Paulo State, Brazil (22°49.886′S/48°25.426′W and 22°54.353′S/48°14.562′W, respectively), in July and October 2015, respectively. Both *At. sexdens rubropilosa* and *M. goeldii* colonies were sampled in shadowed and humid areas of eucalyptus cultivation, with approximately 3–10 m of distance between colonies. Top and bottom sections of fungus gardens were sampled from two colonies of *At. sexdens rubropilosa,* and were combined for resulting 92.59 g. Because of the smaller size of *M. goeldii* fungus gardens, central and peripherical regions were sampled from 18 colonies and were combined for totalizing 50.58 g of fungus garden. Immediately after collection, samples were kept under controlled conditions (25 °C, in the dark) for subsequent preparations.

### Bacterial sampling, DNA extraction and sequencing

Bacterial fractions were obtained from fungus-gardens through a centrifugation and filtration protocol modified from Suen et al*.*^27^ and Aylward et al*.*^28^. Briefly, workers, larvae, and pupae were removed from the samples, and fungus garden were buffered in 1X PBS (137 mM NaCl, 2.7 mM KCl, 10 mM Na_2_HPO_4_, and 2 mM KH_2_PO_4_) containing 0.1% Tween 80 and gently centrifuged (30 min at 40 × g). This mixture was incubated at room temperature for six days for fungus gardens of *At. sexdens rubropilosa* and for ten days for *M. goeldii* gardens. During this period, the fungus garden settled at the bottom of the tubes. The buffer was carefully transferred to another tube, filtered, and centrifuged (30 min at 2,800 × g), then the resulting pellet was stored at 8 °C. The fungus garden was washed in fresh buffer, centrifuged (30 min at 40 × g) and incubated in the same conditions. The washing and incubation steps were repeated three times. Following these washing steps, the mixture was shaken for 3 min, filtered, and centrifuged for 30 min at 2,800 × *g*. Then, the several pellets resulting from the same sample were joined. The presence of bacteria in the final pellet was confirmed through bright-field microscopy. DNA from 0.40 g of sample was subsequently extracted from the bacterial fraction using the PowerSoil DNA Isolation Kit (MoBio Laboratories). We empirically verified this adaptation resulting in DNA samples with higher quantity and quality from our bacterial samples. DNA was sequenced through Illumina HiSeq 2000, paired ending sequencing (100 bp).

### Assembling and annotation of *Mycocepurus goeldii* and *Atta sexdens rubropilosa* metagenomes

Quality control and preprocessing of reads were carried out in Solexa QA v3.1.5^91^, sorting the reads by quality (*phred cutoff* = 13) and length (*length cutoff* = 60). Preprocessing quality was checked in FastQC. Quality-controlled reads were assembled using default settings in MEGAHIT v1.0.6^92^. Quality of assembled contigs were verified in PRINSEQ^93^. Quality-controlled contigs were uploaded to the Integrated Microbial Genomes (IMG) database for gene identification and annotation through the standard pipeline of IMG^94^. Protein-coding sequences were taxonomically assigned using the “Phylogenetic Distribution of Genes” comparative tool of IMG^42^, which estimates the phylogenetic composition of the metagenome by comparing (through RPS-BLAST) the best BLASTp hits with COG database. The taxonomic assignment was performed at bacterial genera level (identity percentage > 60%). Taxonomic classification was further confirmed through two distinct approaches. First, contigs > 100Kbp were taxonomically assigned using PhyloPhytiaS^95^. Second, a phylogenetic analysis was based on protein sequences of eight phylogenetic marker genes: *alaS* (COG0013); *uvrC* (COG0322); *recN* (COG 0,497); *pyrG* (COG0504); *ffh/srp* (COG0541); *uvrB* (COG0556); *radA*, (COG1066); and *typA* (COG1217)^96^. Since there is only a single copy of these genes in most of bacterial genomes, their sequences are considered proper for bacterial taxonomic classification^96,97^. All protein-coding sequences corresponding to the COG functions above mentioned were exported from IMG using the Phylogenetic Marker COGs tool, and aligned to their best BLASTp hits using MUSCLE^98^. Maximum-likelihood phylogenies were inferred through PhyML^99^, using WAG as substitution model and 100 replicates of non-parametric bootstrap analysis.

### Comparative analysis: taxonomic composition, KEGG pathways, and CAZy profile

Metagenomes from *M. goeldii* and *At. sexdens rubropilosa* fungus gardens were grouped to FGI metagenomes from a previous study^36^ for functional and taxonomic comparisons to diverse hosts’ metagenomes publicly available at the IMG database (accessed: April, 2018). For these comparisons, we downloaded 155 host-associated metagenomes publicly available at IMG (Access: March–May, 2018), from phylogenetically related and phylogenetically distant hosts, grouping them by diet and lifestyle as follows: herbivorous insects, omnivorous insects, marine communities (including corals and marine worms), herbivorous vertebrates, and omnivorous vertebrates (please see Supplementary Table S1 for metagenomes’ IMG IDs, details and references on hosts taxonomic and diet classification). Comparisons were based on the relative abundance of protein-coding sequences, i.e., number of sequences annotated for a specific function (or taxa)/total number of protein-coding sequences. Relative abundances were multiplied by 10^6^ for statistical analysis.

Microbiota taxonomic composition was inferred through the taxonomic assignment of protein-coding sequences by the “Phylogenetic Distribution of Genes” comparative tool of IMG^42^, at bacterial class level (identity percentage > 60%). Diversity indices were estimated based on the relative abundance of bacterial class using PAST 3. Similarities in the microbiota composition between hosts with different diets were determined by UPGMA-clustering based on Bray–Curtis distances with Boot N = 10,000, calculated in PAST 3 using the normalized abundance of bacterial class^100^. Further comparisons based on White’s non-parametric *t*-test^105^ (Bonferroni corrected *P* < 0.05) were performed between FGI microbiota and all other hosts using STAMP v2.1.3^101^. Metagenomic clustering was performed through an alignment-free *k-*mer based approach (*k* = 15), described by Fan et al*.*^102^ For resolution improvements, the clustering tree was estimated using samples with k-mer diversity ranging from 20 to 400 million, which resulted in 137 branches (metagenomes). The K-mer based phylogenetic tree was edited using iTOL^103^ https://itol.embl.de/).

The functional profile was estimated through KEGG pathways (via KO terms)^104^ following the IMG pipeline. Comparisons were based on the relative abundance of KO annotated sequences classified as subsets of KEGG pathways. KO annotated sequences were compiled as metabolic pathways, which were subsequently compiled as: carbohydrate metabolism, amino acid metabolism, energy metabolism, lipid metabolism, glycan metabolism, metabolism of cofactors and vitamins, metabolism of terpenoids and polyketides, biosynthesis of other secondary metabolites, and xenobiotics biodegradation and metabolism. The normalized abundance of KEGG assigned sequences were used for inferring the UPGMA-clustering (based on Bray–Curtis distances with Boot N = 10,000). Further comparisons based on White’s non-parametric *t*-test^105^ (Bonferroni corrected *P* < 0.05) were performed between FGI microbiota and all other hosts using STAMP v2.1.3^101^.

Protein-coding sequences were annotated for Carbohydrates-Active Enzymes (CAZy)^106^ by dbCAN2 meta server (access: May, 2018)^107^. CAZy annotation was carried out by integrating the tools HMMER (E-Value < 1e-15, coverage > 0.35), DIAMOND (E-Value < 1e^-102^), and Hotpep (Frequency > 2.6, Hits > 6). Protein coding sequences annotated as CAZy were classified by CAZy family (GH, GT, CE, CBM, AA, and PL) using Blast2GO Pro, and these sequences were taxonomically annotated using GhostKOALA^108^ by searching in ‘genus_prokaryotes and family_eukaryotes’ KEGG GENES database. The relative abundance of CAZy-sequences taxonomically annotated were used for inferring the abundance heatmaps. Comparisons based on Welch’s t-test were carried for detecting similarities in the relative abundance of CAZy sequences taxonomically-assigned between FGI and all other hosts using STAMP v2.1.3^101^. Box plots were calculated based on the normalized abundance of specific CAZy families, which were compared between host groups using the White’s non-parametric *t*-test^108^ in STAMP v2.1.3^101^.

## Data availability

Metagenomes generated in this study are deposited at the IMG database, under the IMG Genome IDs 3300009856 (*M. goeldii* fungus garden) and 3300009944 (*At. sexdens rubropilosa* fungus garden). IMG ID of publicly available metagenomes also used in this study are listed in the Supplementary Material.

## Supplementary information

Supplementary table S3

Supplementary table S1

Supplementary information
