# Supplementary material for: Fungus-growing insects host a distinctive microbiota apparently adapted to the fungiculture environment
Source: Sci Rep. 2020 Jul 24;10:12384. doi: 10.1038/s41598-020-68448-7 (PMC7381635; doi:10.1038/s41598-020-68448-7)
Supplement: Supplementary file 3 — Supplementary information [file 41598_2020_68448_MOESM3_ESM.pdf]

Fungus-growing insects host a distinctive microbiota apparently adapted to the fungiculture environment

Mariana O Barcoto, Camila Carlos-Shanley, Huan Fan, Milene Ferro, Nilson S Nagamoto, Mauricio Bacci Jr., Cameron Currie, André Rodrigues

Supplementary material

Table S2- Metagenome assembly and annotation statistics for *Mycocepurus goeldii* and *Atta sexdens rubropilosa* microbiota

	M. goeldii	*A. sexdens rubropilosa*
Number of contigs	267,959	139,603
Size of assembled data (Mbp)	364.1	249.6
Mean contig length (bp)[1]	1358.97	1788.20
Largest contig (kbp) [1]	1040.6	716.7
Mean GC content	49.45 ± 12.58 %	57.53 ± 8.14 %
N50 contig size (bp) [1]	4 324	5 495
Contigs > 100 kbp	229	153
Number of annotated sequences	151,313	82,170
Protein coding genes	395,806	269,449
COG annotation (%)	62.62	75.17
Pfam annotation (%)	67.82	76.45
KO annotation (%)	44.03	54.32
KEGG Enzymes annotation (%)	23.60	26.73

[1]Quality and length of assembled contigs are considered good according to Ghurye *et al.*[1]

Table S4 – Classification of CAZy families and KEGG Pathways abundantly codified by fungus-growing insects' microbiota

**Pathways**	**Function**	**Metabolic role**	**References**
**Plant biomass metabolism (Lignocellulose metabolism)**	Beta glucosidase GH1<br>Beta glucosidase GH3	Beta glucosidase in GH1 and GH3 families degrade the oligosaccharides resulting from endo- and exo-cleavage of cellulose to glucose	van den Brink *et al.*[2]
	GH43 Beta xylosidase	Involved in hemicellulose depolimerization by hydrolyzing xylan-oligosaccharides and releasing xylose molecules	Bosetto *et al.*[3]
	Manganese peroxidase AA2	Catalyzes the oxidation of Mn(II) to Mn(III), which oxidizes several phenolic compounds that degrade or modify lignin	Levasseur *et al.*[4]
	Cellobiose dehydrogenase AA3	Cellulose, hemicellulose and lignin depolimerization via oxidation of cellodextrins	Levasseur *et al.*[4]
	Iron reductase AA8	Generates highly reactive hydroxyl radicals which could be involved in nonenzymatic cellulose breakdown	Levasseur *et al.*[4]
	Propanoate and Butanoate metabolism (KEGG Pathways)	Propionate and butyrate are short-chain fatty acids produced by anaerobic fermentation of soluble plant fiber	White *et al.*[5]<br>Louis *et al.*[6]
	Benzoate metabolism (KEGG)	Benzoate is an intermediate in aromatic compounds metabolism. Pathways of benzoate metabolism may be involved both in detoxification of benzoate-derived defensive allelochemicals and in lignin degradation	Takabayashi *et al.*[7]<br>Cheng *et al.*[8]
**Plant biomass metabolism (Detoxification of plant secondary compounds - PSC)**	Geraniol degradation (KEGG)	Pathway involved in plant terpenes detoxification also codified by the gut microbiota of herbivorous hosts	Jing *et al.*[9]

	Limonene and pinene degradation (KEGG)	Pathways promoting detoxification of plant terpenoid metabolites, usually codified by the microbiota of herbivorous feeding on toxic tissues, as woodrats, pine weevils, and mountain pine beetles	Khol *et al.*[10]<br>Berasategui *et al.*[11]<br>Adams *et al.*[12]
	Glutathione metabolism (KEGG)	Pathway participant in detoxifying several stress related compounds, including the conversion of plant glucosinolate-derived isothiocyanates to glutathione conjugates	Masip *et al.*[13]<br>Schramm *et al.*[14]
	Sulfur metabolism (KEGG Pathways)	Involved in plant toxins detoxification via cyanide-detoxifying enzymes, as sulfurtransferases	Burrow *et al.*[15]<br>Zhu *et al.*[16]
	Glyoxylate and dicarboxylate metabolism (KEGG)	Metabolites in the glyoxylate and dicarboxylate pathway seems to be involved in plant defense, though the specific mechanisms are not clear	Hubbard *et al.*[17]
**Plant biomass metabolism (Simpler carbohydrates metabolism)**	Alpha amylase GH13	Catalyzes starch degradation	Maier *et al.*[18]
	Starch phosphorylase GT35	Amylolytic enzyme	Warren *et al.*[19]
	Sucrose synthase GT4	Reversibly catalyzes the conversion of sucrose and a nucleoside diphosphate into fructose and nucleotide (NDP)-glucose	Diricks *et al.*[20]
**Biofilm formation**	Cellulose synthase GT2	Synthesis of bacterial cellulose, a structural component of biofilms which facilitates bacterial-host interactions	Augimeri *et al.*[21]<br>Serra *et al.*[22]
	Chitin deacetylase CE4	Deacetylation of extracellular polysaccharides are required for biofilm formation	Aragunde *et al.*[23]

	N-acetylglucosamine deacetylase CE9<br><br>N -acetylglucosamine 6 phosphate deacetylase CE11	Exopolysaccharides (as N-acetylglucosamine) are the main component in biofilm matrix. Postsynthetic deacetylation of exopolysaccharides is required to biofilm formation for enhancing adhere to host cells or abiotic surfaces	Lee *et al.*[24]<br>Ostapska *et al.*[25]
	Lipopolysaccharide biosynthesis (KEGG)	Host surface's microbiome tend to abundantly codifies lipopolysaccharide biosynthesis genes	Ofek-Lalzar *et al.*[26]<br>Sivadon *et al.*[27]
	Biofilm and plant biomass degradation	Biofilm optimize lignocellulolytic activity and assess the detoxification of plant secondary compounds (PSC)	Leng *et al.*[28]<br>Macfarlane *et al.*[29]
	Biofilm and fungal biomass metabolism	Biofilm mediates fungal-microbiota interactions (including the mycolytic activity of some bacteria) by facilitating aggregation around the hyphae, and ultimately the hyphae surface colonization	Deveau *et al.*[30]
**Fungal biomass metabolism**	Chitinase GH23	Cleavage of glycosidic linkages in chitin and chitodextrins, resulting in chitooligosaccharides. Chitinase activity, that seems to be induced by chitin-derived oligomers, destabilize and degrade fungal cell walls	Deveau *et al.*[30]<br>Montgomery *et al.*[31]
	Chitin deacetylase CE4	Required for biofilm formation, which is important for chitin surface colonization	Aragunde *et al.*[23]<br>Sivadon *et al.*[27]
	Chitin binding modules CBM50	Chitin-binding proteins allow bacterial cells attachment to the chitin surface, ensuring efficient chitinolytic activity and colonization	Montgomery *et al.*[31]

References may be found at the end of this file.

Figure S1- Taxonomic assignment of sequences > 100Kbp. A) *Mycocepurus goeldii* microbiota. B) *Atta sexdens rubropilosa* microbiota.

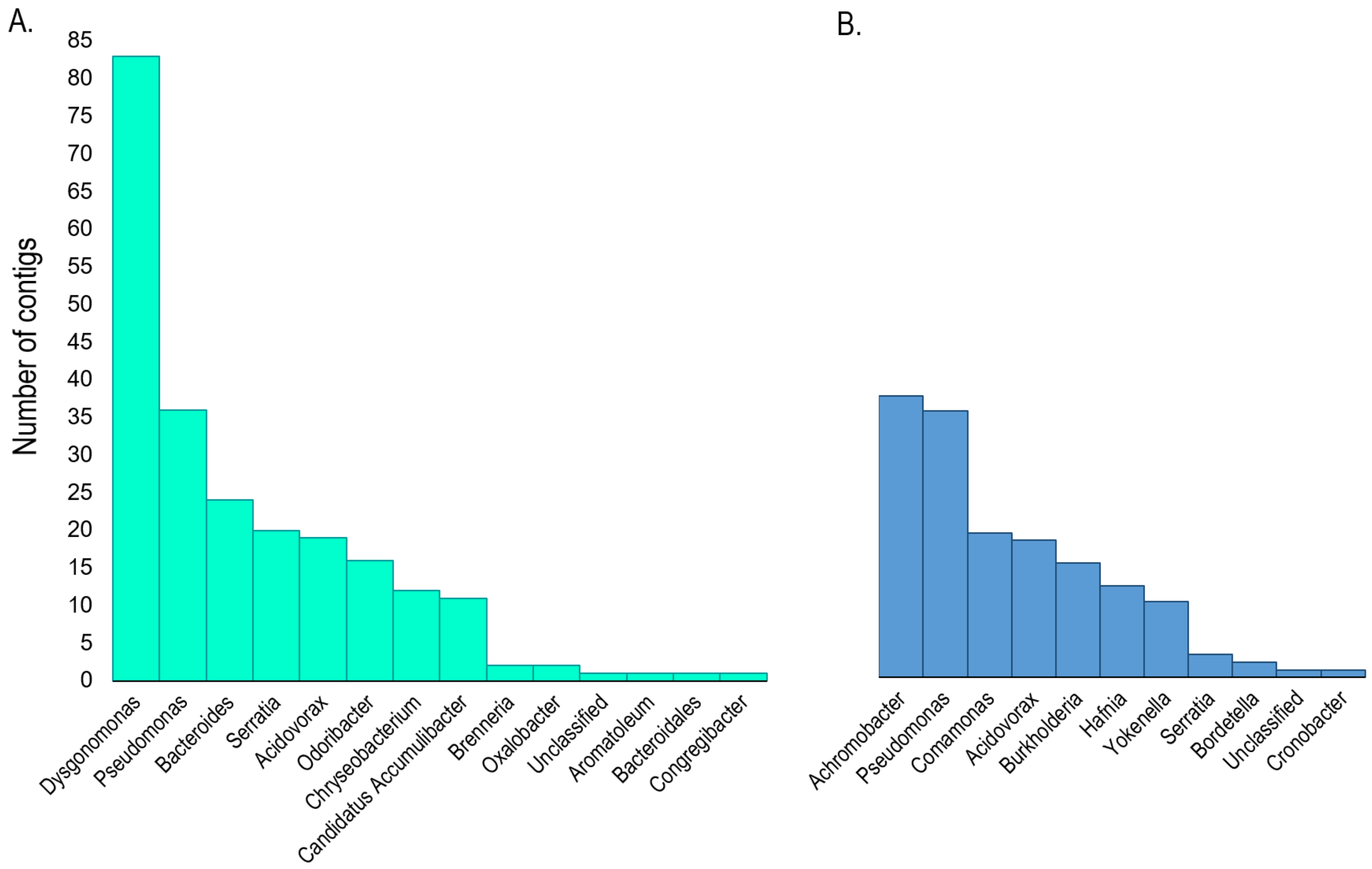

Figure S2- Bacterial genera in the microbiota of *Mycocepurus goeldii* and *Atta sexdens rubropilosa* fungus gardens, predicted by metagenome aligning against the phylogenetic marker genes *radA* (COG1066) and *typA* (COG1217). In bold are the bacterial genera to which metagenomic contigs were aligned.

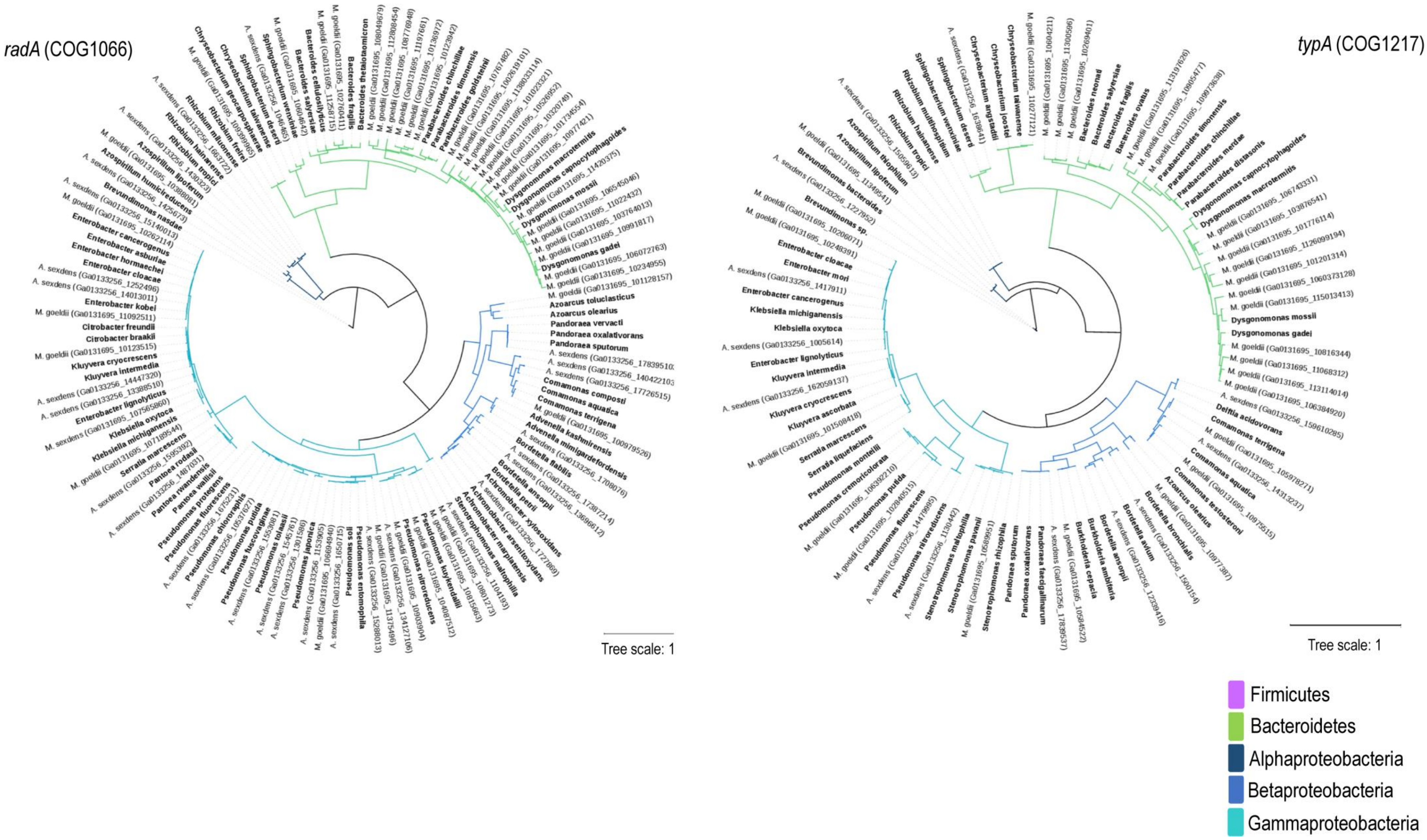

Figure S3- Bacterial genera in the microbiota of *Mycocepurus goeldii* and *Atta sexdens rubropilosa* fungus gardens, predicted by metagenome aligning against the phylogenetic marker genes *ffh/srp* (COG0541) and *uvrB* (COG0556). In bold are the bacterial genera to which metagenomic contigs were aligned.

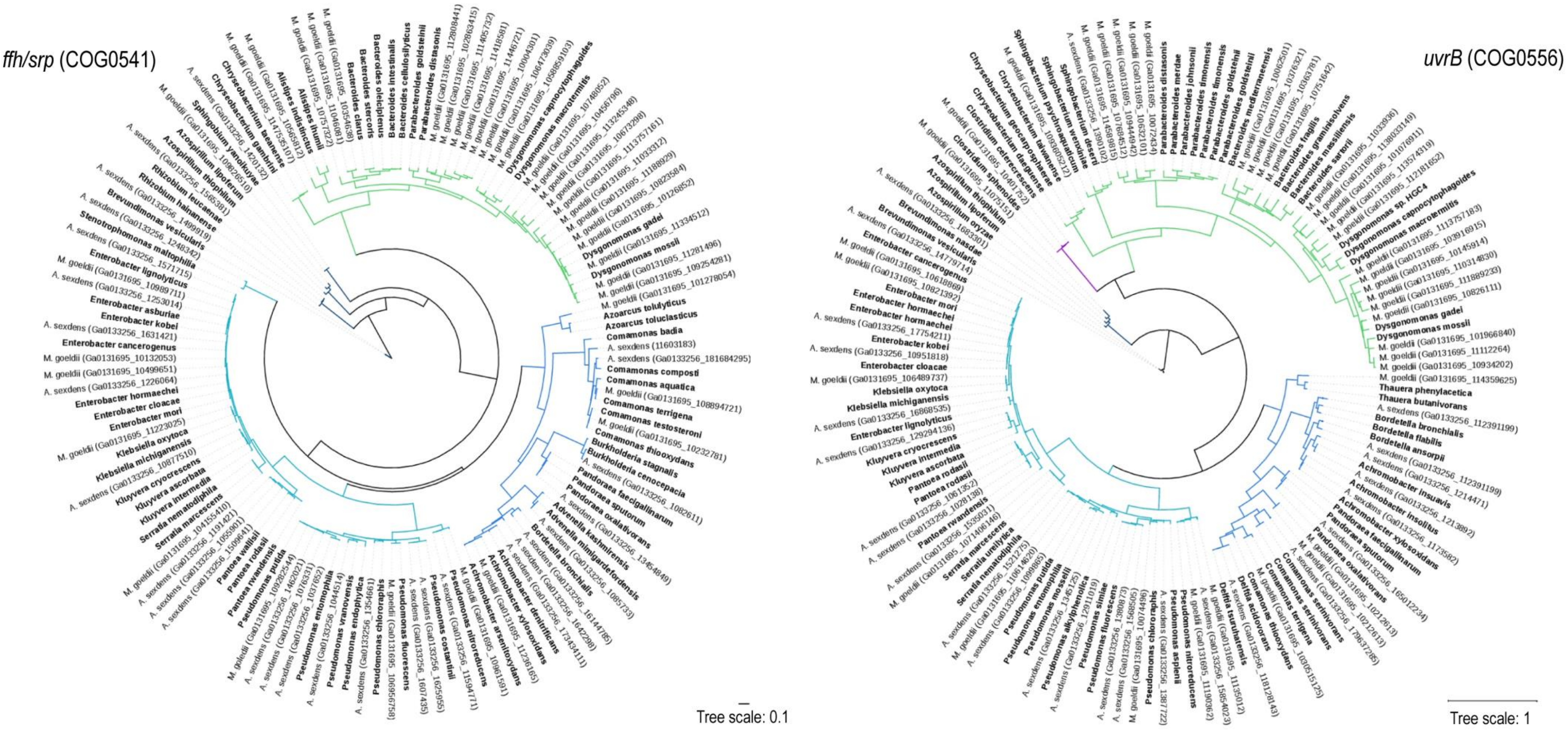

Figure S4- Bacterial genera in the microbiota of *Mycocepurus goeldii* and *Atta sexdens rubropilosa* fungus gardens, predicted by metagenome aligning against the phylogenetic marker genes *recN* (COG 0497) and *pyrG* (COG0504). In bold are the bacterial genera to which metagenomic contigs were aligned.

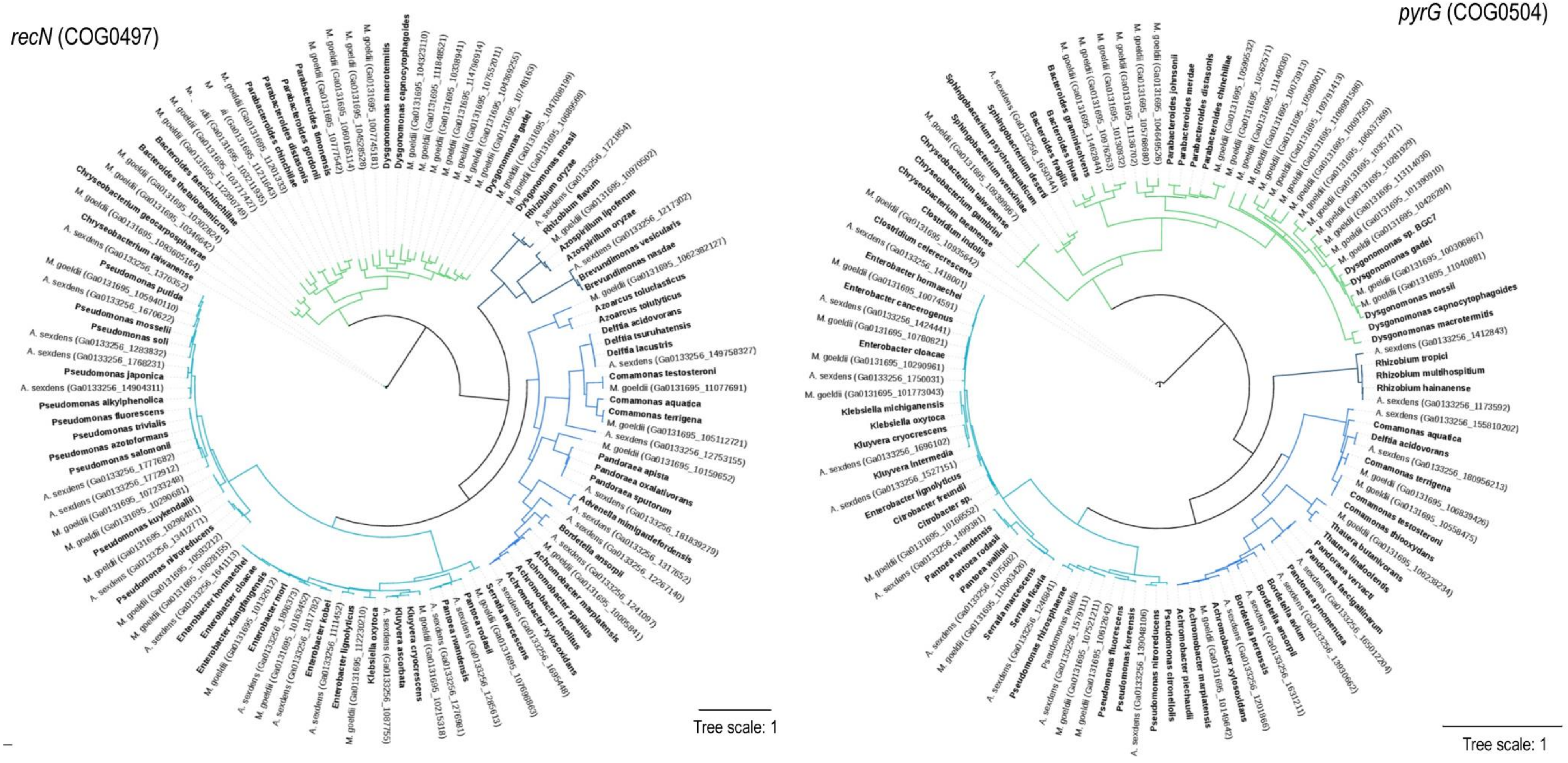

Figure S5- Bacterial genera in the microbiota of *Mycocepurus goeldii* and *Atta sexdens rubropilosa* fungus gardens, predicted by metagenome aligning against the phylogenetic marker genes *alaS* (COG0013) and *uvrC* (COG0322). In bold are the bacterial genera to which metagenomic contigs were aligned.

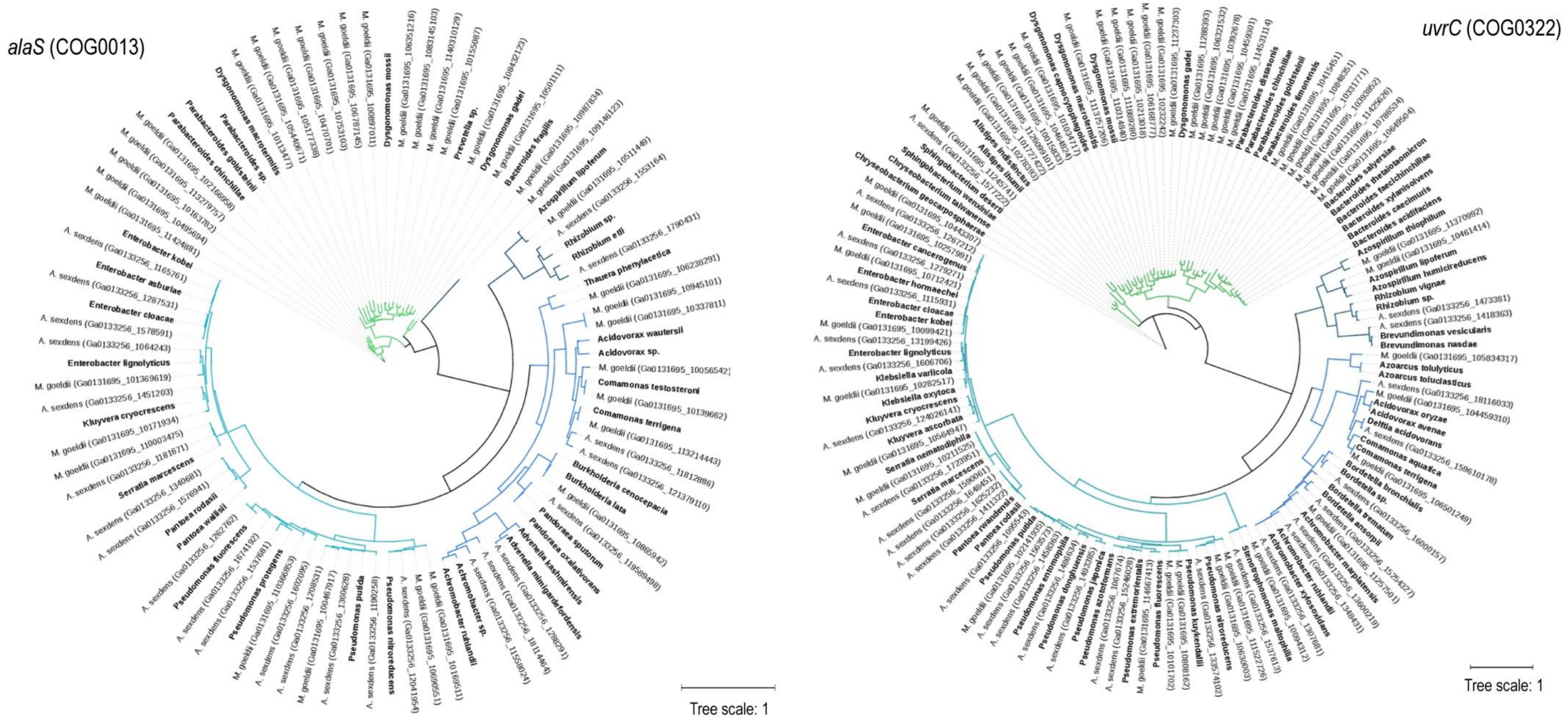

Figure S6- Microbiota composition. Comparisons between the microbiota of fungus-growing insects and other hosts and herbivorous insects. All comparisons were performed using the relative abundance of taxonomically assigned protein-coding sequences. Significant differences (White test, Bonferroni corrected *P*<0.05) in bacterial classes between: **A.** Fungus-growing and herbivorous insects; **B.** Fungus-growing insects and herbivorous vertebrates **C.** Fungus-growing insects and omnivorous vertebrates; **D.** Fungus-growing insects and marine communities. No significant differences were found between fungus-growing and omnivorous insects.

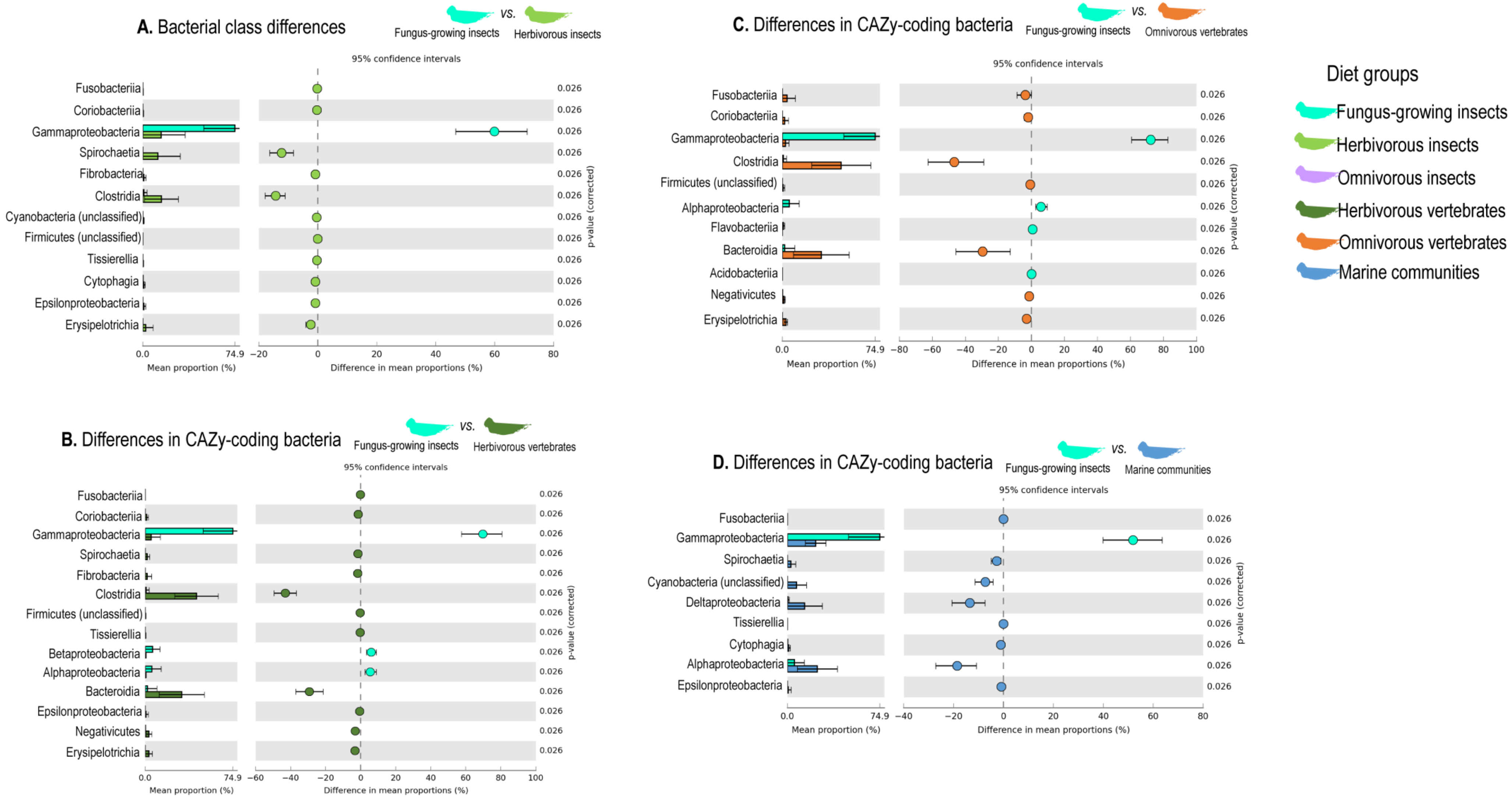

Figure S7- Microbiota diversity at class level Diversity indices **A.** Taxa richness **B.** Shannon indices (H). **C.** Dominance indices (D). **D.** Evenness indices (e^H/S) estimated based on the relative abundance of bacterial class, depicted as box plots according to the hosts' diet group and phylogeny. Double-colored boxes represent a phylogenetic group having hosts with more than one type of diet (e.g., herbivorous and omnivorous hosts in the same phylogenetic group).

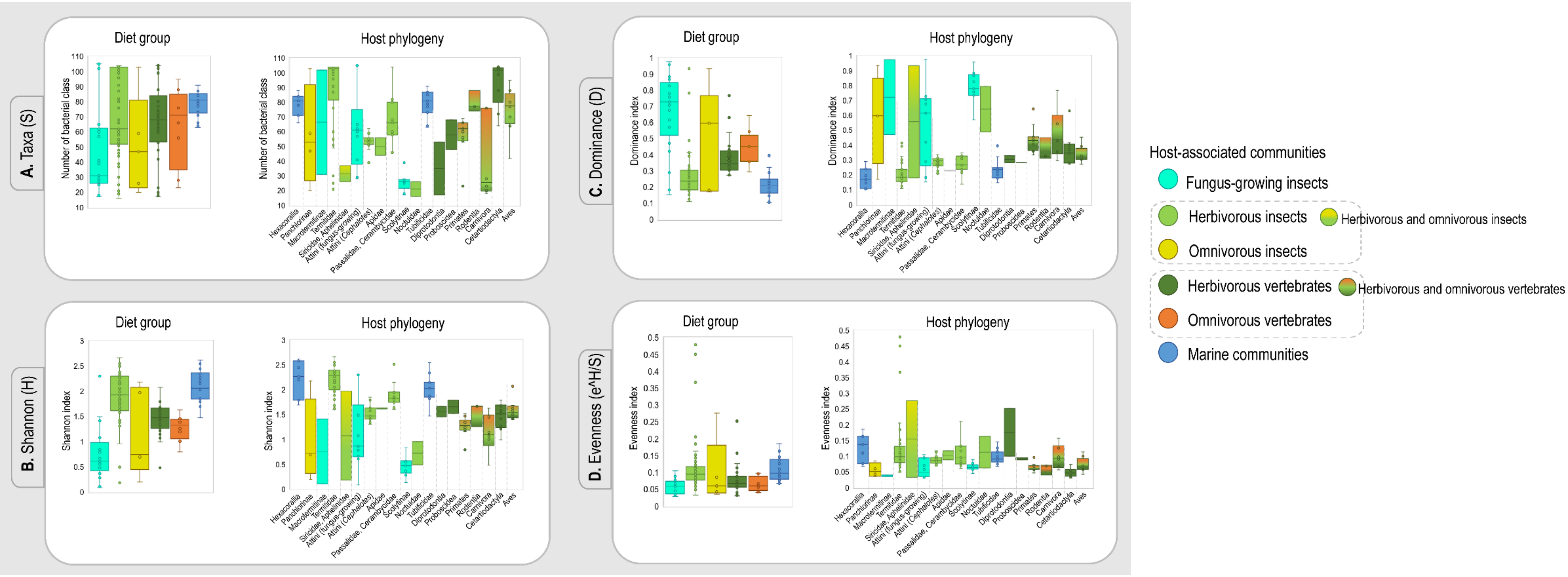

Figure S8- CAZy-codifier bacterial groups Comparisons between fungus-growing systems and other hosts for CAZy-codifier members of the microbiota. Comparisons were performed using the relative abundance of taxonomically-assigned CAZy sequences. Significant differences (Welch's t-test, Bonferroni corrected $P<0.05$) in bacterial classes between the microbiota of: **A.** Fungus-growing and herbivorous insects; **B.** Fungus-growing and omnivorous insects; **C.** Fungus-growing insects and herbivorous vertebrates; **D.** Fungus-growing insects and omnivorous vertebrates; **E.** Fungus-growing insects and marine communities; **F.** Macrotermitinae termites and Scolytinae beetles; **G.** Macrotermitinae and other Termitidae termites; **H.** Fungus-growing attini ants and *Cephalotes* ants; **I.** Scolytinae and Passalidae / Cerambycidae beetles.

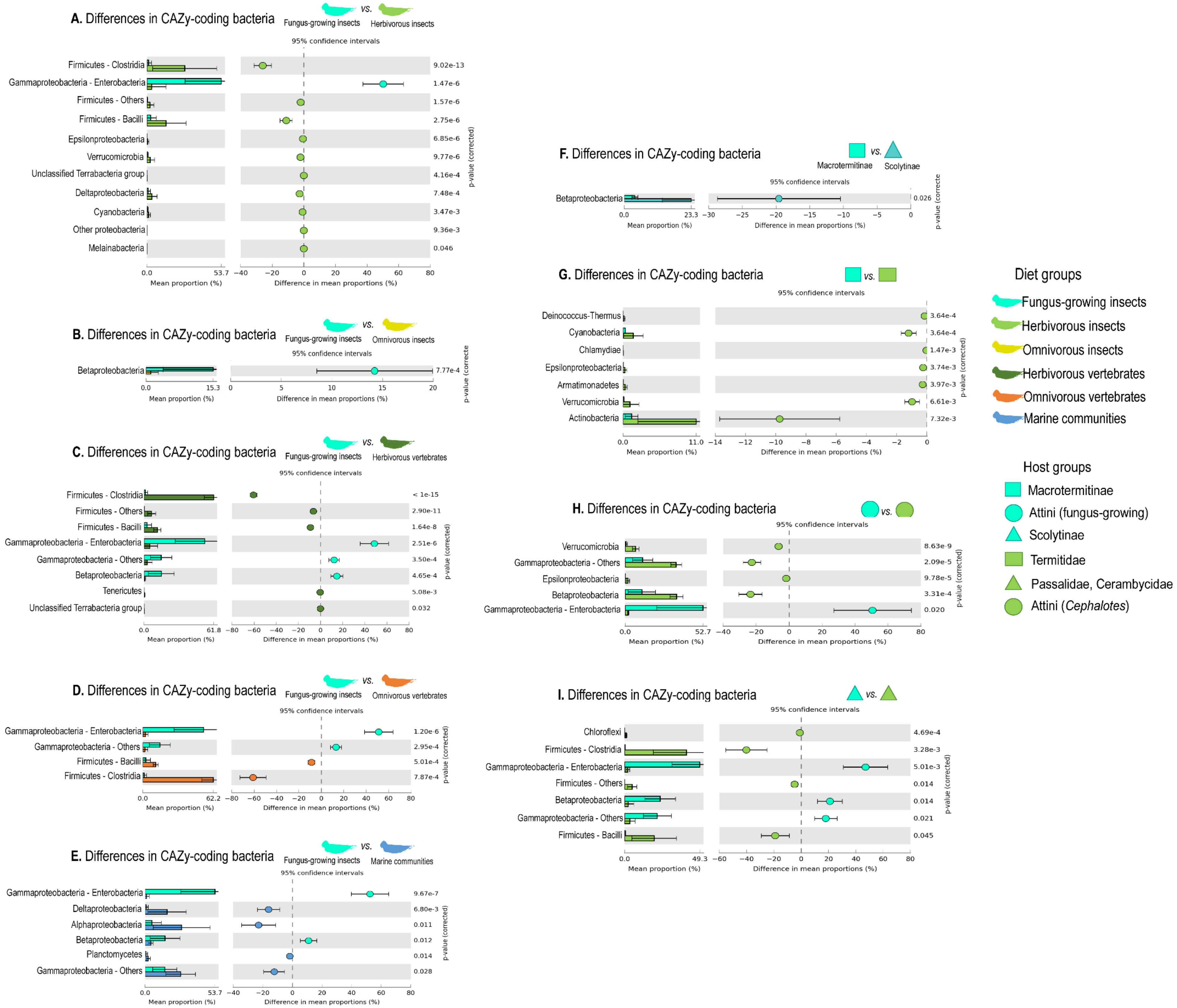
A. Differences in CAZy-coding bacteria
Fungus-growing insects vs. Herbivorous insects
95% confidence intervals
Firmicutes - Clostridia 9.02e-13
Gammaproteobacteria - Enterobacteria 1.47e-6
Firmicutes - Others 1.57e-6
Firmicutes - Bacilli 2.75e-6
Epsilonproteobacteria 6.85e-6
Verrucomicrobia 9.77e-6
Unclassified Terrabacteria group 4.16e-4
Deltaproteobacteria 7.48e-4
Cyanobacteria 3.47e-3
Other proteobacteria 9.36e-3
Melainabacteria 0.046
0.0 53.7 Mean proportion (%)
−40 −20 0 20 40 60 80 Difference in mean proportions (%)
p-value (corrected)
B. Differences in CAZy-coding bacteria
Fungus-growing insects vs. Omnivorous insects
95% confidence intervals
Betaproteobacteria 7.77e-4
0.0 15.3 Mean proportion (%)
0 5 10 15 20 Difference in mean proportions (%)
p-value (correcte
C. Differences in CAZy-coding bacteria
Fungus-growing insects vs. Herbivorous vertebrates
95% confidence intervals
Firmicutes - Clostridia < 1e-15
Firmicutes - Others 2.90e-11
Firmicutes - Bacilli 1.64e-8
Gammaproteobacteria - Enterobacteria 2.51e-6
Gammaproteobacteria - Others 3.50e-4
Betaproteobacteria 4.65e-4
Tenericutes 5.08e-3
Unclassified Terrabacteria group 0.032
0.0 61.8 Mean proportion (%)
−80 −60 −40 −20 0 20 40 60 80 Difference in mean proportions (%)
p-value (corrected)
D. Differences in CAZy-coding bacteria
Fungus-growing insects vs. Omnivorous vertebrates
95% confidence intervals
Gammaproteobacteria - Enterobacteria 1.20e-6
Gammaproteobacteria - Others 2.95e-4
Firmicutes - Bacilli 5.01e-4
Firmicutes - Clostridia 7.87e-4
0.0 62.2 Mean proportion (%)
−80 −60 −40 −20 0 20 40 60 80 Difference in mean proportions (%)
p-value (corrected)
E. Differences in CAZy-coding bacteria
Fungus-growing insects vs. Marine communities
95% confidence intervals
Gammaproteobacteria - Enterobacteria 9.67e-7
Deltaproteobacteria 6.80e-3
Alphaproteobacteria 0.011
Betaproteobacteria 0.012
Planctomycetes 0.014
Gammaproteobacteria - Others 0.028
0.0 53.7 Mean proportion (%)
−40 −20 0 20 40 60 80 Difference in mean proportions (%)
p-value (corrected)
F. Differences in CAZy-coding bacteria
Macrotermitinae vs. Scolytinae
95% confidence intervals
Betaproteobacteria 0.026
0.0 23.3 Mean proportion (%)
−30 −25 −20 −15 −10 −5 0 Difference in mean proportions (%)
p-value (correcte
G. Differences in CAZy-coding bacteria
vs.
95% confidence intervals
Deinococcus-Thermus 3.64e-4
Cyanobacteria 3.64e-4
Chlamydiae 1.47e-3
Epsilonproteobacteria 3.74e-3
Armatimonadetes 3.97e-3
Verrucomicrobia 6.61e-3
Actinobacteria 7.32e-3
0.0 11.0 Mean proportion (%)
−14 −12 −10 −8 −6 −4 −2 0 Difference in mean proportions (%)
p-value (corrected)
H. Differences in CAZy-coding bacteria
vs.
95% confidence intervals
Verrucomicrobia 8.63e-9
Gammaproteobacteria - Others 2.09e-5
Epsilonproteobacteria 9.78e-5
Betaproteobacteria 3.31e-4
Gammaproteobacteria - Enterobacteria 0.020
0.0 52.7 Mean proportion (%)
−40 −20 0 20 40 60 80 Difference in mean proportions (%)
p-value (corrected)
I. Differences in CAZy-coding bacteria
vs.
95% confidence intervals
Chloroflexi 4.69e-4
Firmicutes - Clostridia 3.28e-3
Gammaproteobacteria - Enterobacteria 5.01e-3
Firmicutes - Others 0.014
Betaproteobacteria 0.014
Gammaproteobacteria - Others 0.021
Firmicutes - Bacilli 0.045
0.0 49.3 Mean proportion (%)
−60 −40 −20 0 20 40 60 80 Difference in mean proportions (%)
p-value (corrected)
Diet groups
Fungus-growing insects
Herbivorous insects
Omnivorous insects
Herbivorous vertebrates
Omnivorous vertebrates
Marine communities
Host groups
Macrotermitinae
Attini (fungus-growing)
Scolytinae
Termitidae
Passalidae, Cerambycidae
Attini (*Cephalotes*)

Figure S9 - Most abundant CAZy-codifier members. Relative abundance of the most abundant CAZy-codifier classes, following the hosts' clusters observed in main Fig. 3. **A.** Gammaproteobacteria (Enterobacteria). **B.** Gammaproteobacteria (Others). **C.** Betaproteobacteria. **D.** Alphaproteobacteria. **E.** Firmicutes (Bacilli). **F.** Firmicutes (Clostridia). **E.** Bacteroidetes. **F.** Actinobacteria. **G.** Spirochaetes.

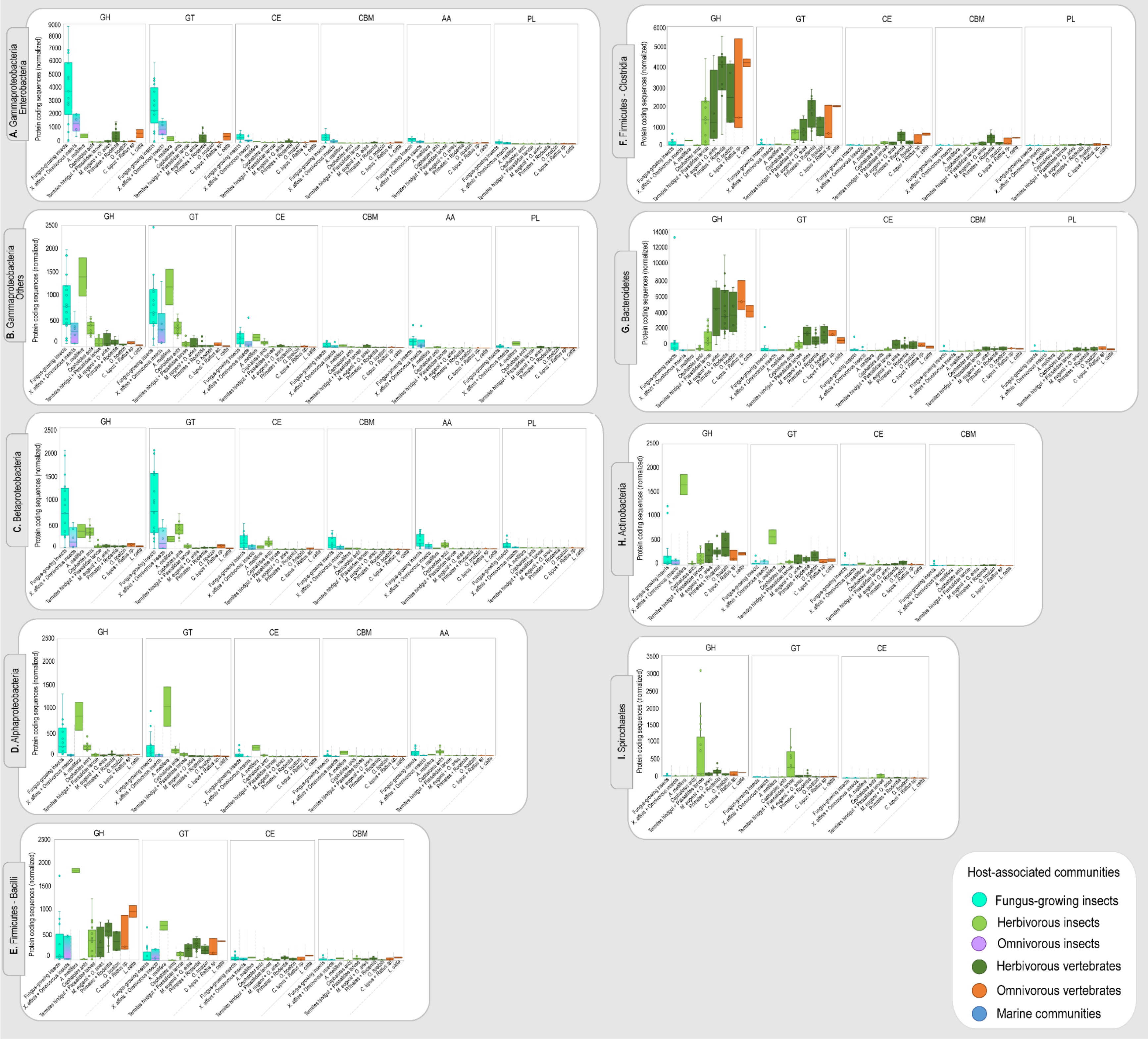
A. Gammaproteobacteria Enterobacteria
B. Gammaproteobacteria Others
C. Betaproteobacteria
D. Alphaproteobacteria
E. Firmicutes - Bacilli
F. Firmicutes - Clostridia
G. Bacteroidetes
H. Actinobacteria
I. Spirochaetes
GH
GT
CE
CBM
AA
PL
Protein coding sequences (normalized)
Host-associated communities
Fungus-growing insects
Herbivorous insects
Omnivorous insects
Herbivorous vertebrates
Omnivorous vertebrates
Marine communities

Figure S10- Carbohydrate metabolism Comparisons between the microbiota of fungus-growing insects and other hosts for KEGG pathways of carbohydrate metabolism. Comparisons were performed using the relative abundance of KO annotated sequences classified as pathways of carbohydrate metabolism. **A.** KEGG pathways that compose carbohydrate metabolism. Significant differences (White test, Bonferroni corrected *P*<0.05) in KEGG pathways between: **B.** Fungus-growing and herbivorous insects; **C.** Fungus-growing insects and herbivorous vertebrates; **D.** Fungus-growing insects and omnivorous vertebrates; **E.** Fungus-growing insects and marine communities. No significant differences were found between fungus-growing and omnivorous insects.

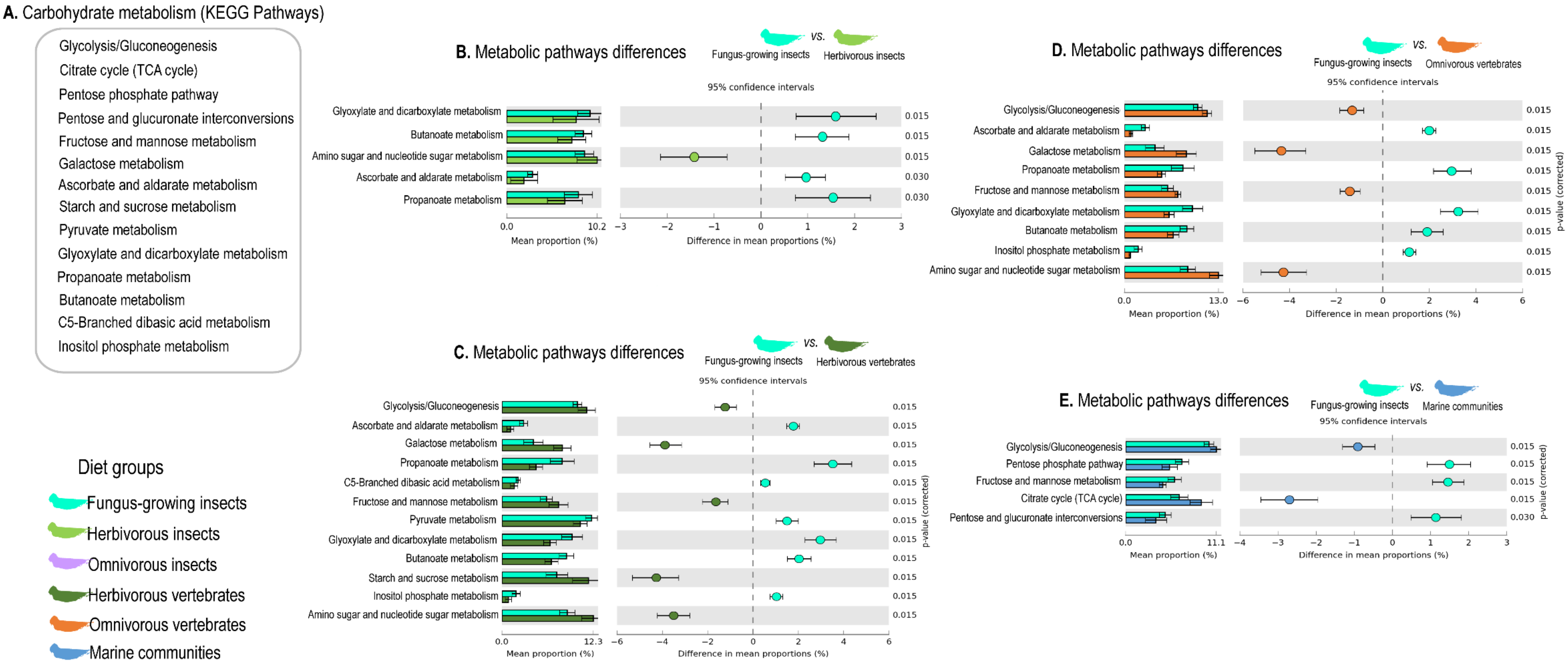

Figure S11- Amino acid metabolism Comparisons between the microbiota of fungus-growing insects and other hosts for KEGG pathways of amino acid metabolism. Comparisons were performed using the relative abundance of KO annotated sequences classified as pathways of amino acid metabolism. **A.** KEGG pathways that compose amino acid metabolism. Significant differences (White test, Bonferroni corrected *P*<0.05) in KEGG pathways between: **B.** Fungus-growing and herbivorous insects; **C.** Fungus-growing insects and omnivorous insects; **D.** Fungus-growing insects and herbivorous vertebrates; **E.** Fungus-growing insects and omnivorous vertebrates; **F.** Fungus-growing insects and marine communities.

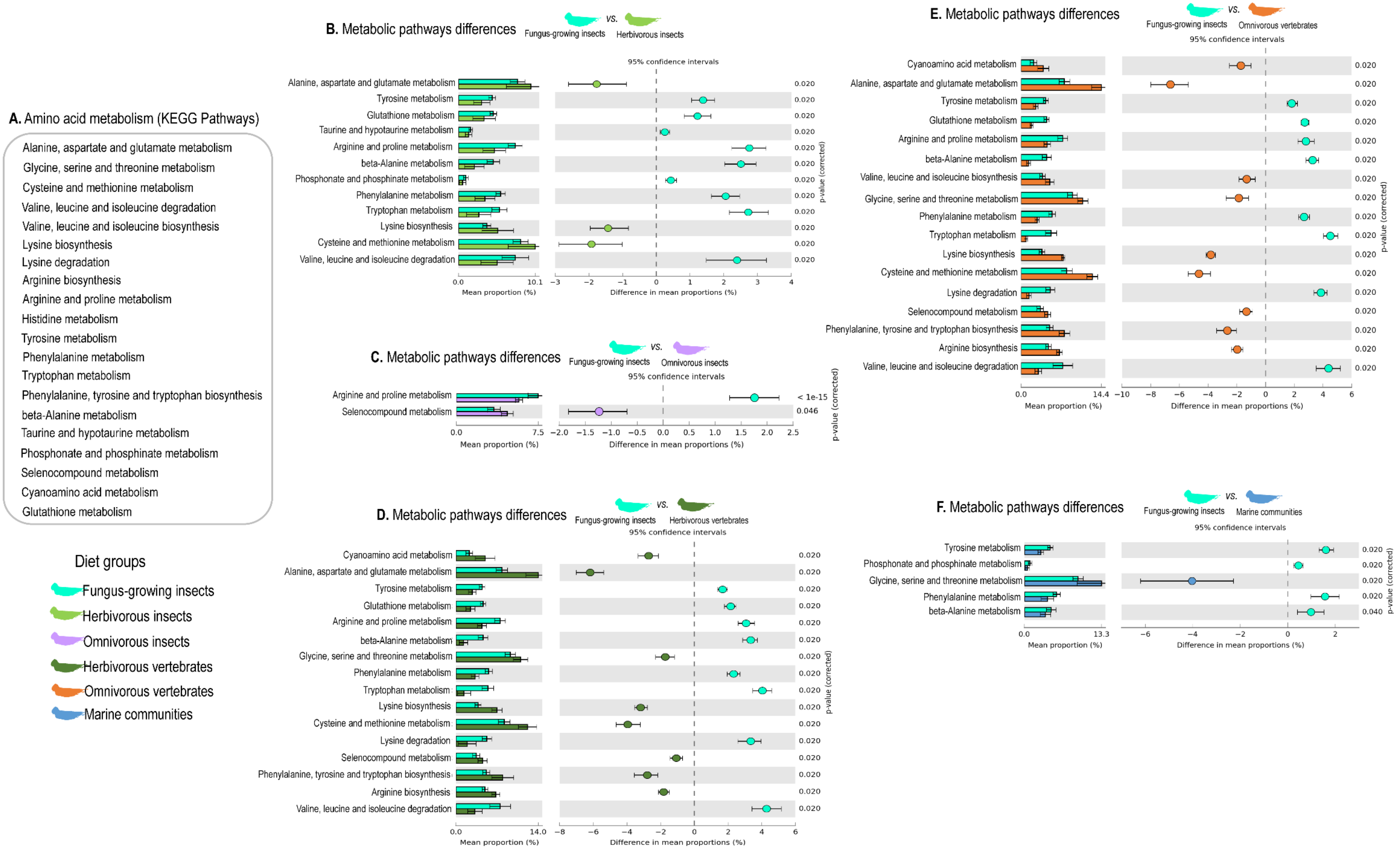

Figure S12- Energy metabolism Comparisons between the microbiota of fungus-growing insects and other hosts for KEGG pathways of energy metabolism. Comparisons were performed using the relative abundance of KO annotated sequences classified as KEGG pathways of energy metabolism. **A.** KEGG pathways that compose energy metabolism. Significant differences (White test, Bonferroni corrected $P<0.05$) in KEGG pathways between: **B.** Fungus-growing and herbivorous insects; **C.** Fungus-growing insects and herbivorous vertebrates; **D.** Fungus-growing insects and omnivorous vertebrates; **E.** Fungus-growing insects and marine communities. No significant differences were found between fungus-growing and omnivorous insects.

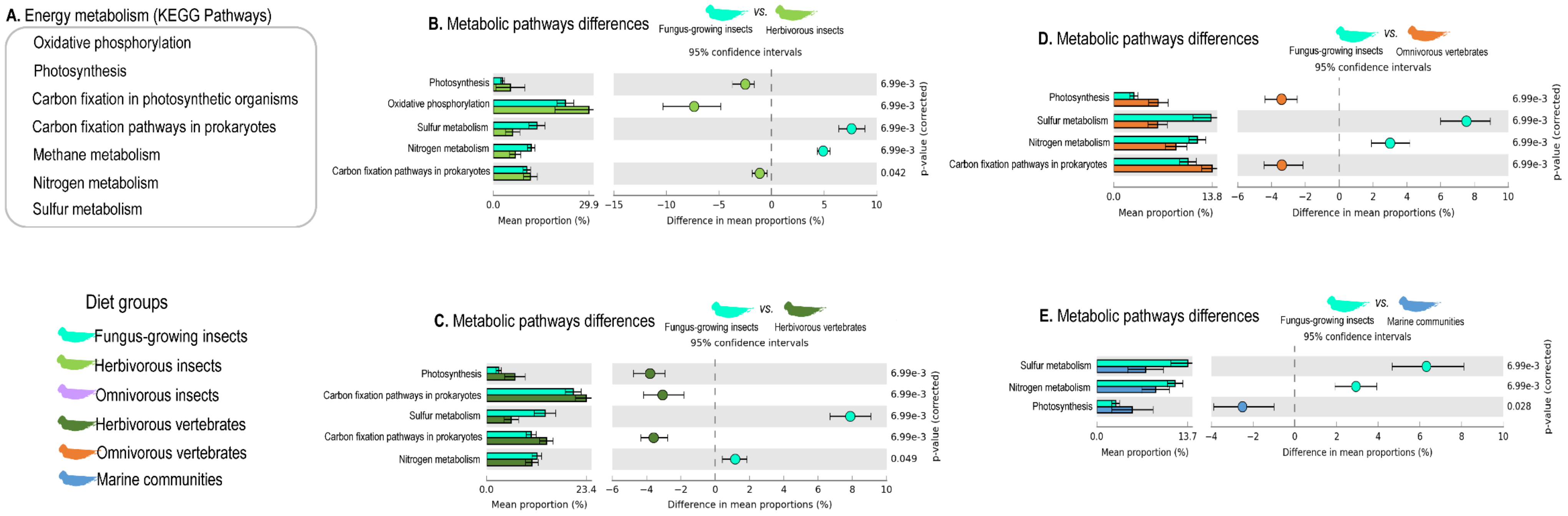

Figure S13- Glycan biosynthesis and metabolism Comparisons between the microbiota of fungus-growing insects and other hosts for KEGG pathways of glycan metabolism. Comparisons were performed using the relative abundance of KO annotated sequences classified as KEGG pathways of glycan metabolism. **A.** KEGG pathways that compose glycan metabolism. Significant differences (White test, Bonferroni corrected *P*<0.05) in KEGG pathways between: **B.** Fungus-growing and herbivorous insects; **C.** Fungus-growing insects and herbivorous vertebrates; **D.** Fungus-growing insects and omnivorous vertebrates; **E.** Fungus-growing insects and marine communities. No significant differences were found between fungus-growing and omnivorous insects.

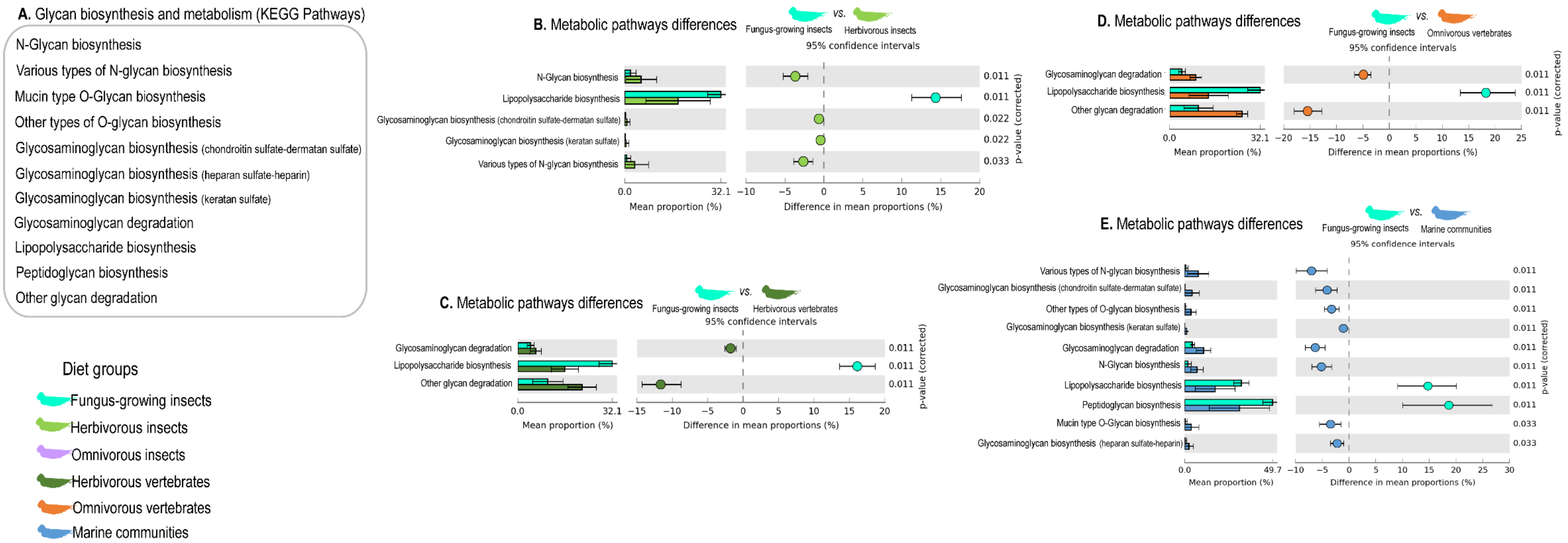

Figure S14- Lipid metabolism Comparisons between the microbiota of fungus-growing insects and other hosts for KEGG pathways of lipid metabolism. Comparisons were performed using the relative abundance of KO annotated sequences classified as pathways of lipid metabolism. **A.** KEGG pathways that compose lipid metabolism. Significant differences (White test, Bonferroni corrected *P*<0.05) in KEGG pathways between: **B.** Fungus-growing and herbivorous insects; **C.** Fungus-growing insects and herbivorous vertebrates; **D.** Fungus-growing insects and omnivorous vertebrates; **E.** Fungus-growing insects and marine communities. No significant differences were found between fungus-growing and omnivorous insects.

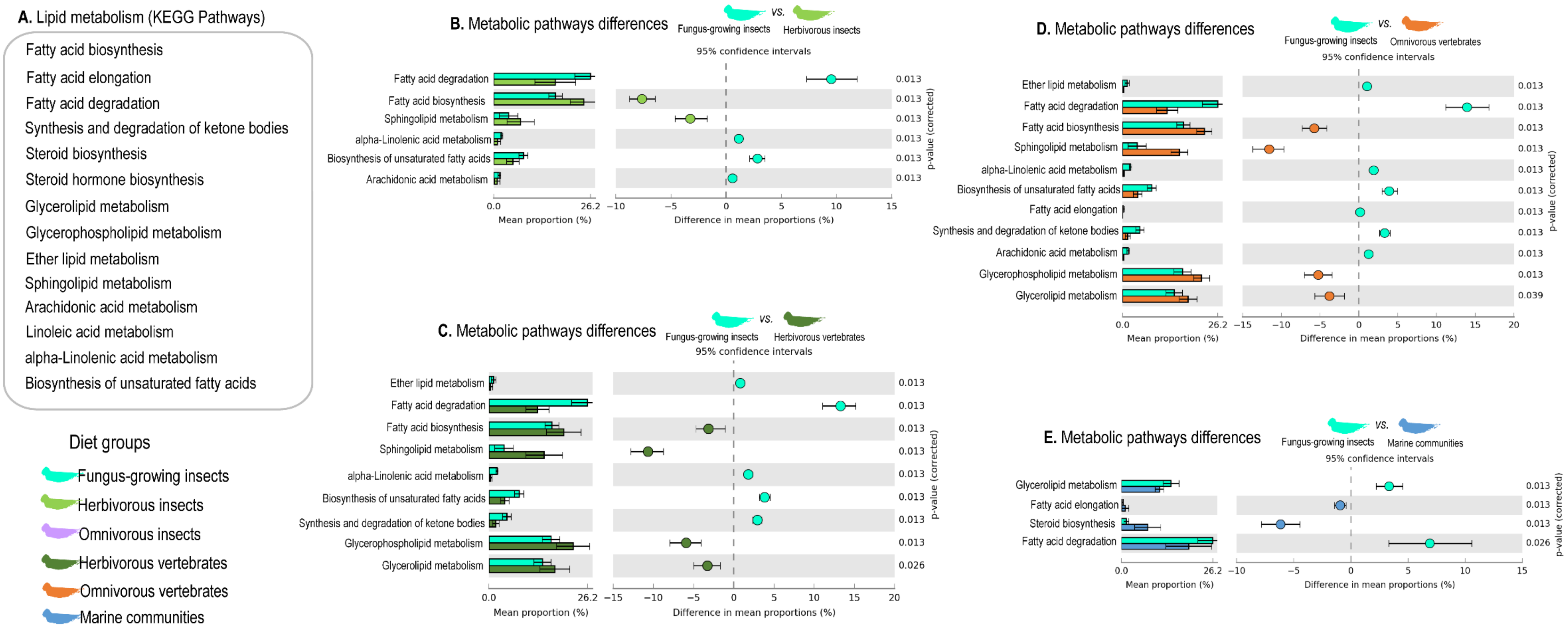

Figure S15- Metabolism of cofactors and vitamins Comparisons between the microbiota of fungus-growing insects and other hosts for KEGG pathways of cofactors and vitamins metabolism. Comparisons were performed using the relative abundance of KO annotated sequences classified as pathways of cofactors and vitamins metabolism. **A.** KEGG pathways that compose cofactors and vitamins metabolism. Significant differences (White test, Bonferroni corrected *P*<0.05) in KEGG pathways between: **B.** Fungus-growing and herbivorous insects; **C.** Fungus-growing insects and herbivorous vertebrates; **D.** Fungus-growing insects and omnivorous vertebrates; **E.** Fungus-growing insects and marine communities. No significant differences were found between fungus-growing and omnivorous insects.

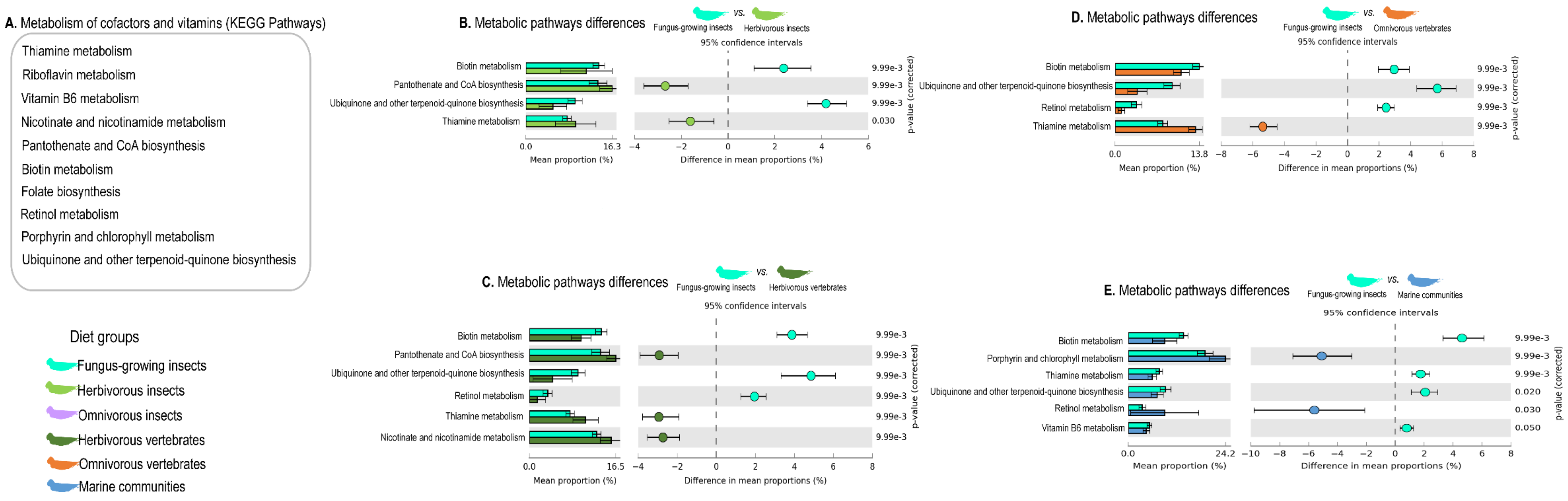

Figure S16- Metabolism of terpenoids and polyketides Comparisons between the microbiota of fungus-growing insects and other hosts for KEGG pathways of terpenoids and polyketides metabolism. Comparisons were performed using the relative abundance of KO annotated sequences classified as pathways of terpenoids and polyketides metabolism. **A.** KEGG pathways that compose terpenoids and polyketides metabolism. Significant differences (White test, Bonferroni corrected *P*<0.05) in KEGG pathways between: **B.** Fungus-growing and herbivorous insects; **C.** Fungus-growing insects and omnivorous insects; **D.** Fungus-growing insects and herbivorous vertebrates; **E.** Fungus-growing insects and omnivorous vertebrates; **F.** Fungus-growing insects and marine communities.

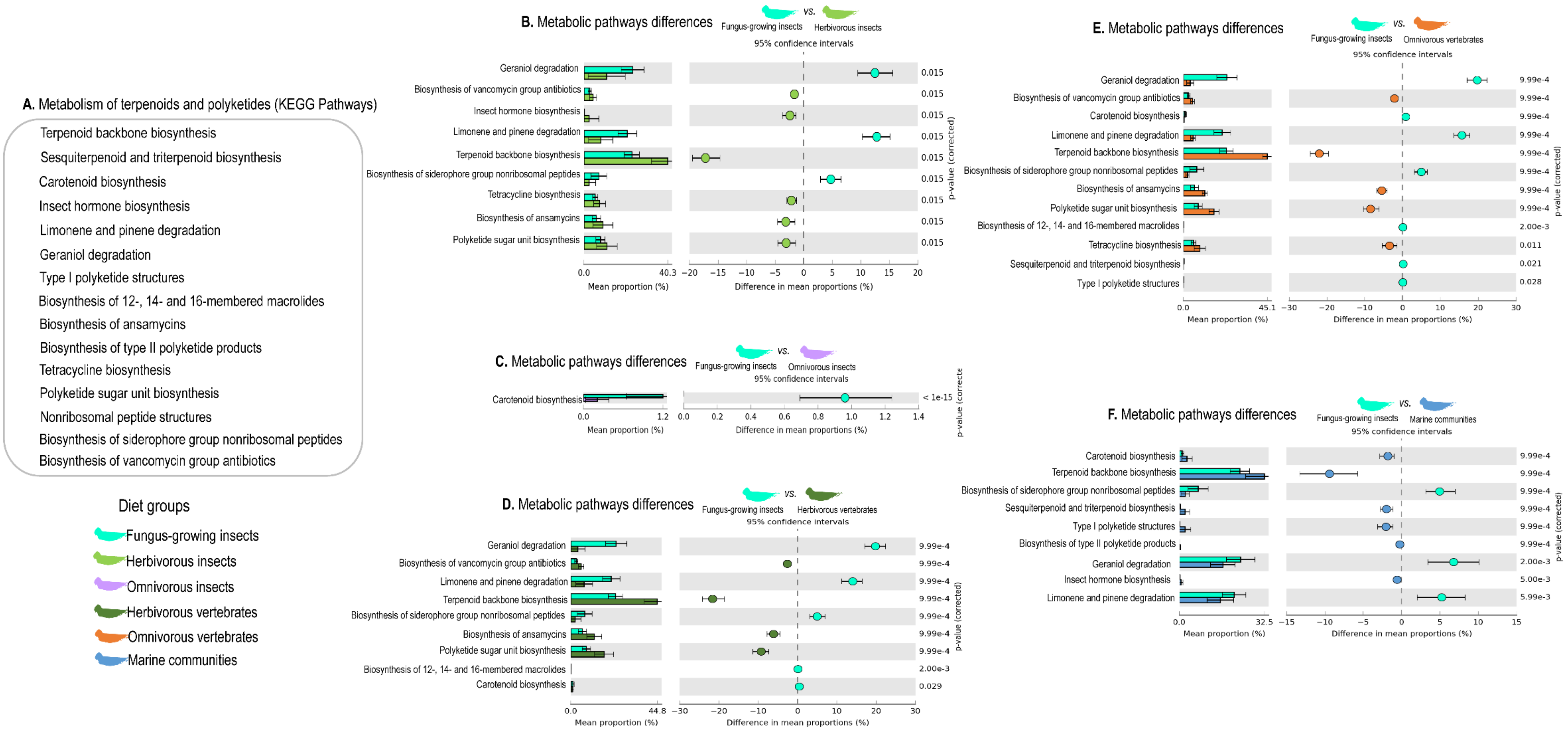

Figure S17- Biosynthesis of other secondary metabolites Comparisons between the microbiota of fungus-growing insects and other hosts for KEGG pathways of biosynthesis of other secondary metabolites. Comparisons were performed using the relative abundance of KO annotated sequences classified as pathways of biosynthesis of other secondary metabolites. **A.** KEGG pathways that compose biosynthesis of other secondary metabolites. Significant differences (White test, Bonferroni corrected *P*<0.05) in KEGG pathways between: **B.** Fungus-growing and herbivorous insects; **C.** Fungus-growing insects and herbivorous vertebrates; **D.** Fungus-growing insects and omnivorous vertebrates; **E.** Fungus-growing insects and marine communities No significant differences were found between fungus-growing and omnivorous insects.

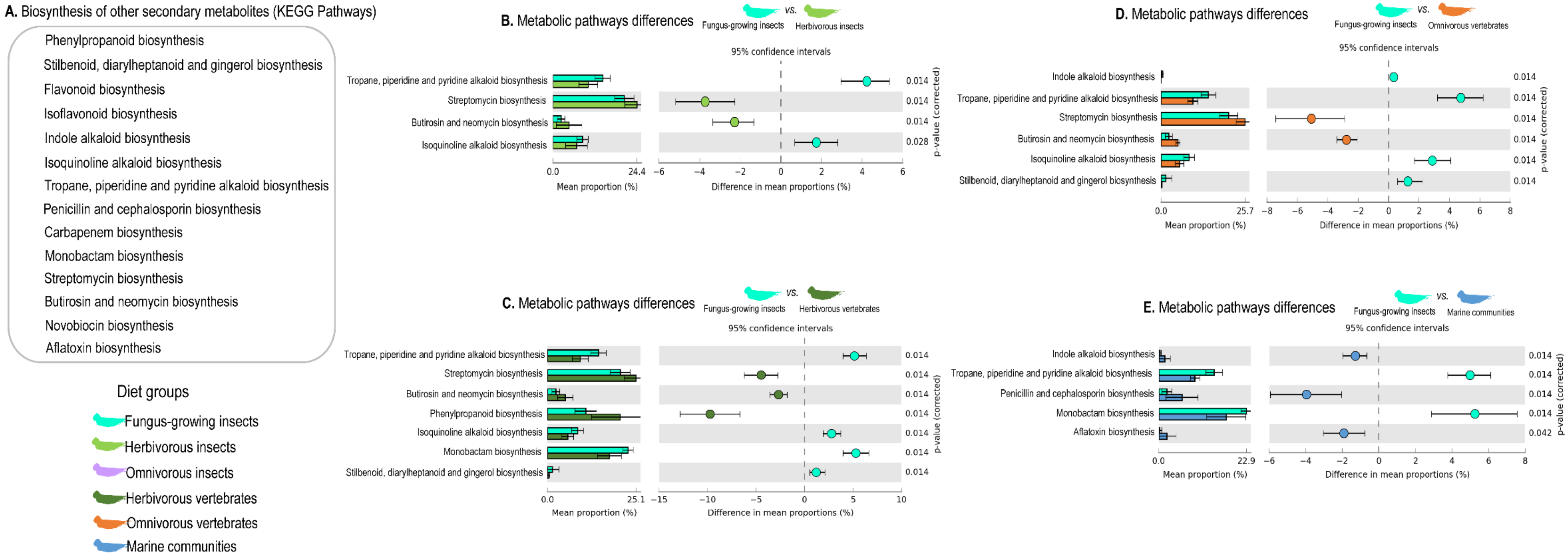

Figure S18- Xenobiotics biodegradation and metabolism Comparisons between the microbiota of fungus-growing insects and other hosts for KEGG pathways of xenobiotics metabolism. Comparisons were performed using the relative abundance of KO annotated sequences classified as pathways of xenobiotics metabolism. **A.** KEGG pathways that compose xenobiotics metabolism. Significant differences (White test, Bonferroni corrected P<0.05) in KEGG pathways between: **B.** Fungus-growing and herbivorous insects; **C.** Fungus-growing insects and omnivorous insects; **D.** Fungus-growing insects and herbivorous vertebrates; **E.** Fungus-growing insects and omnivorous vertebrates; **F.** Fungus-growing insects and marine communities.

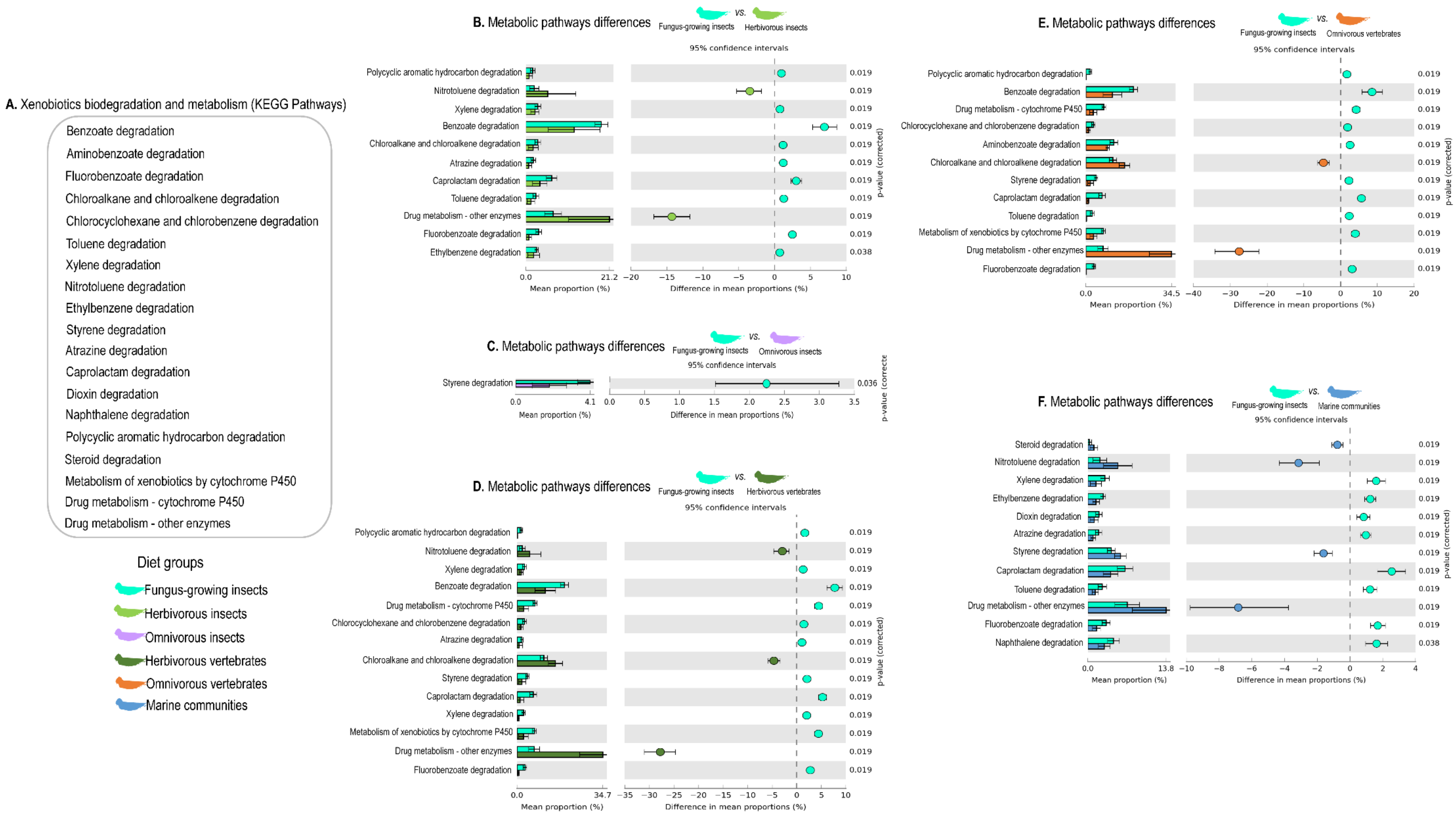

Supplementary References

1. Ghurye, J. S., Cepeda-Espinoza, V. & Pop, M. Focus: microbiome: metagenomic assembly: Overview, Challenges and Applications. *Yale J. Biol. Med.*, **89**, 353–362 (2016).

2. van den Brink, J. & de Vries, R.P. Fungal enzyme sets for plant polysaccharide degradation. *Appl. Microbiol. Biotechno.l* **91,** 1477–1492 (2011). https://doi.org/10.1007/s00253-011-3473-2

3. Bosetto, A. *et al.* Research Progress Concerning Fungal and Bacterial β-Xylosidases. *Appl. Biochem. Biotechnol.* **178,** 766–795 (2016). https://doi.org/10.1007/s12010-015-1908-4

4. Levasseur, A. *et al.* Expansion of the enzymatic repertoire of the CAZy database to integrate auxiliary redox enzymes. *Biotechnol. Biofuels* **6,** 41 (2013). https://doi.org/10.1186/1754-6834-6-41

5. White, B.A., Lamed, R., Bayer, E.A., *&* Flint, H.J. Biomass utilization by gut microbiomes. *Annu. Rev. Microbiol.* **68,** 279-296 (2014).

6. Louis, P. & Flint, H. J. Formation of propionate and butyrate by the human colonic microbiota. *Environ. Microbiol.* **19**(1), 29-41 (2017).

7. Takabayashi, J., Dicke, M. & Posthumus, M.A. Volatile herbivore-induced terpenoids in plant-mite interactions: Variation caused by biotic and abiotic factors. *J. Chem. Ecol.* **20,** 1329–1354 (1994). https://doi.org/10.1007/BF02059811

8. Cheng, X., Tian, X. *et al.* Metagenomic analysis of the pinewood nematode microbiome reveals a symbiotic relationship critical for xenobiotics degradation. *Sci. Rep.* **3,** 1869 (2013). https://doi.org/10.1038/srep01869

9. Jing, T., Qi, F. & Wang, Z. Most dominant roles of insect gut bacteria: digestion, detoxification, or essential nutrient provision? *Microbiome* **8,** 38 (2020). https://doi.org/10.1186/s40168-020-00823-y

10. Kohl, K. D. *et al.* Metagenomic sequencing provides insights into microbial detoxification in the guts of small mammalian herbivores (*Neotoma* spp.). *FEMS Microbiol. Ecol.*, **94(**12), fiy184 (2018).

11. Berasategui, A. *et al.* Gut microbiota of the pine weevil degrades conifer diterpenes and increases insect fitness. *Mol. Ecol.* **26**(15), 4099-4110 (2017). https://doi.org/10.1111/mec.14186

12. Adams, A. S. *et al.* Mountain pine beetles colonizing historical and naïve host trees are associated with a bacterial community highly enriched in genes contributing to terpene metabolism. *Appl. Environ. Microbiol.* **79**, 3468–3475 (2013).

13. Masip, L., Veeravalli, K. & Georgiou, G. The many faces of glutathione in bacteria. *Antioxidants & redox signaling* **8**(5-6), 753-762 (2006). https://doi.org/10.1089/ars.2006.8.753

14. Schramm, K., Vassão, D. G., Reichelt, M., Gershenzon, J., & Wittstock, U. Metabolism of glucosinolate-derived isothiocyanates to glutathione conjugates in generalist lepidopteran

herbivores. *Insect Biochem. Mol. Biol.*, **42**(3), 174-182 (2012). https://doi.org/10.1016/j.ibmb.2011.12.002

15. Burow M., Wittstock U. & Gershenzon J. Sulfur-Containing Secondary Metabolites and Their Role in Plant Defense. [Hell R., Dahl C., Knaff D., Leustek T. (eds)] *Sulfur Metabolism in Phototrophic Organisms. Advances in Photosynthesis and Respiration*, Vol 27, 201-222. (Springer, Dordrecht, 2008).

16. Zhu, L. *et al.* Potential mechanism of detoxification of cyanide compounds by gut microbiomes of bamboo-eating pandas. *MSphere*, **3**(3) (2018). https://doi.org/10.1128/mSphere.00229-18.

17. Hubbard, C. J. *et al.* The effect of rhizosphere microbes outweighs host plant genetics in reducing insect herbivory. *Mol. Ecol.* **28**(7), 1801-1811 (2019). https://doi.org/10.1111/mec.14989

18. Maier, T. V. *et al.* Impact of dietary resistant starch on the human gut microbiome, metaproteome, and metabolome. *mBio* **8**, e01343-17 (2017). https://doi.org/10.1128/mBio.01343-17

19. Warren, F. J. *et al.* Food starch structure impacts gut microbiome composition. *Msphere* **3**, e00086-18 (2018). https://doi.org/10.1128/mSphere.00086-18

20. Diricks, M. *et al.* Identification of sucrose synthase in nonphotosynthetic bacteria and characterization of the recombinant enzymes. *Appl. Microbiol. Biotechnol*. **99**, 8465–8474 (2015). https://doi.org/10.1007/s00253-015-6548-7

21. Augimeri, R. V., Varley, A. J. & Strap, J. L. Establishing a role for bacterial cellulose in environmental interactions: lessons learned from diverse biofilm-producing Proteobacteria. *Front. Microbiol.*, *6*, 1282 (2015). https://doi.org/10.3389/fmicb.2015.01282

22. Serra, D. O., Richter, A. M. & Hengge, R. Cellulose as an architectural element in spatially structured *Escherichia coli* biofilms. *J. Bacteriol.*, **195**, 5540-5554 (2013). https://doi.org/10.1128/JB.00946-13

23. Aragunde, H., Biarnés, X. & Planas, A. Substrate recognition and specificity of chitin deacetylases and related family 4 carbohydrate esterases. *Int. J. Mol. Sci.* **19**, 412 (2018). https://doi.org/10.3390/ijms19020412

24. Lee, M. J. *et al.* Deacetylation of fungal exopolysaccharide mediates adhesion and biofilm formation. *MBio*, **7**, e00252-16 (2016). https://doi.org/10.1128/mBio.00252-16

25. Ostapska, H., Howell, P. L. & Sheppard, D. C. Deacetylated microbial biofilm exopolysaccharides: It pays to be positive. *PLoS Pathog.* **14**(12): e1007411 (2018). doi: 10.1371/journal.ppat.1007411

26. Ofek-Lalzar, M., Sela, N., Goldman-Voronov, M. *et al.* Niche and host-associated functional signatures of the root surface microbiome. *Nat. Commun.* **5,** 4950 (2014). https://doi.org/10.1038/ncomms5950

27. Sivadon, P., Barnier, C., Urios, L. & Grimaud, R. Biofilm formation as a microbial strategy to assimilate particulate substrates. *Environ. Microbiol. Rep.* **11**(6), 749-764 (2019). https://doi.org/10.1111/1758-2229.12785

28. Leng, R. A. Biofilm compartmentalisation of the rumen microbiome: modification of fermentation and degradation of dietary toxins. *Anim. Prod. Sci.*, **57**(11), 2188-2203 (2017).

29. Macfarlane, S. & Macfarlane, G. T. Composition and metabolic activities of bacterial biofilms colonizing food residues in the human gut. *Appl. Environ. Microbiol.*, **72**(9), 6204-6211 (2006). https://doi.org/10.1128/AEM.00754-06

30. Deveau, A. *et al.* Bacterial–fungal interactions: ecology, mechanisms and challenges. *FEMS Microbiol. Rev.* **42**(3), 335-352 (2018). https://doi.org/10.1093/femsre/fuy008

31. Montgomery, M. T. & Kirchman, D. L. Induction of chitin-binding proteins during the specific attachment of the marine bacterium Vibrio harveyi to chitin. *Appl. Environ. Microbiol.* **60**(12), 4284-4288 (1994).
